# Hydrological and soil physiochemical variables determine the rhizospheric microbiota in subtropical lakeshore areas

**DOI:** 10.7717/peerj.10078

**Published:** 2020-09-29

**Authors:** Xiaoke Zhang, Huili Wang, Zhifei Li, Jun Xie, Jiajia Ni

**Affiliations:** 1 Research Center of Aquatic Organism Conservation and Water Ecosystem Restoration in University of Anhui Province, Anqing Normal University, Anqing, China; 2 Key Laboratory of Tropical and Subtropical Fishery Resource Application and Cultivation, Pearl River Fisheries Research Institute, Chinese Academy of Fishery Sciences, Guangzhou, China; 3 Department of Hepatobiliary Surgery II, Guangdong Provincial Research Center of Artificial Organ and Tissue Engineering, Zhujiang Hospital of Southern Medical University, Guangzhou, China; 4 Dongguan Key Laboratory of Medical Bioactive Molecular Developmental and Translational Research, Guangdong Medical University, Dongguan, Guangdong, China

**Keywords:** Lakeshore area, Hydrology, Rhizospheric microbiota, Microbial community

## Abstract

**Background:**

Due to intensive sluice construction and other human disturbances, lakeshore vegetation has been destroyed and ecosystems greatly changed. Rhizospheric microbiota constitute a key part of a functioning rhizosphere ecosystem. Maintaining rhizosphere microbial diversity is a central, critical issue for sustaining these rhizospheric microbiota functions and associated ecosystem services. However, the community composition and abiotic factors influencing rhizospheric microbiota in lakeshore remain largely understudied.

**Methods:**

The spatiotemporal composition of lakeshore rhizospheric microbiota and the factors shaping them were seasonally investigated in three subtropical floodplain lakes (Lake Chaohu, Lake Wuchang, and Lake Dahuchi) along the Yangtze River in China through 16S rRNA amplicon high-throughput sequencing.

**Results:**

Our results showed that four archaeal and 21 bacterial phyla (97.04 ± 0.25% of total sequences) dominated the rhizospheric microbiota communities of three lakeshore areas. Moreover, we uncovered significant differences among rhizospheric microbiota among the lakes, seasons, and average submerged depths. The Acidobacteria, Actinobacteria, Bacteroidetes, Bathyarchaeota, Gemmatimonadetes, and Proteobacteria differed significantly among the three lakes, with more than half of these dominant phyla showing significant changes in abundance between seasons, while the DHVEG-6, Ignavibacteriae, Nitrospirae, Spirochaetes, and Zixibacteria varied considerably across the average submerged depths (*n* = 58 sites in total). Canonical correspondence analyses revealed that the fluctuation range of water level and pH were the most important factors influencing the microbial communities and their dominant microbiota, followed by total nitrogen, moisture, and total phosphorus in soil. These results suggest a suite of hydrological and soil physiochemical variables together governed the differential structuring of rhizospheric microbiota composition among different lakes, seasons, and sampling sites. This work thus provides valuable ecological information to better manage rhizospheric microbiota and protect the vegetation of subtropical lakeshore areas.

## Introduction

Rhizospheric microbiota (RM) is a vital component of the rhizosphere ecosystem in lakeshore areas. The interaction of plant roots with innumerable microbial communities within this niche has a considerable impact on developmental stages of lakeshore plants and their tolerance to stressful conditions (Shilev et al., 2001; Dennis, Miller & Hirsch, 2010; Gill et al., 2016; Bandyopadhyay et al., 2017). For instance, higher plant growth and biomass were obtained when bacteria selected from metal-contaminated soil were added to experimental soil (Shilev et al., 2001). Plant-associated microbes are considered as “helpers” that can provide additional genes to the host for acclimatization of the latter in changing or distinctive environmental conditions (Vandenkoornhuyse et al., 2015), although they also associated with soil-borne microbial diseases (Mao et al., 2019; Mao et al., 2020). Recently, the recruitment of RM into the rhizosphere gained much attention among researchers (Standing et al., 2005; Bandyopadhyay et al., 2017; Zhang et al., 2019; Kavamura et al., 2020). Related studies with terrestrial plants have shown that the factors influencing microbial recruitment include plant genotype and age, edaphic factors, geographical location, and climatic changes (Berg & Smalla, 2009; Bulgarelli et al., 2012; Tkacz et al., 2015; Bandyopadhyay et al., 2017). But given the enormous species diversity of plants and microbes, and the staggering number of potential interactions and complex community structure within the rhizosphere, our understanding about the drivers of this recruitment process is still uncovered. To better understand it, one must distinguish the microbial species and influencing factors that contribute to the formation of complex RM.

The lakeshore is an important ecological ecotone between terrestrial and aquatic ecosystems. As the primary producer and main constituent of lakeshore habitats, lakeshore plants play vital roles in maintaining the structural and functional stability of lake ecosystems. For this reason, lake managers often strive to conserve and create greenbelts in lakeshore areas threatened by eutrophication and intensive sluice construction (Coops, Vulink & Van Nes, 2004; Zhang et al., 2016; Baastrup-Spohr et al., 2017). Considering the non-trivial benefit of RM for lakeshore plant growth and health, studies on the formation and maintenance mechanisms of RM are very valuable to protect and restore the vegetation of lakeshore areas. However, most research on this topic tends to focus on plant composition and biodiversity (Van Geest et al., 2005; Bayley & Guimond, 2008), standing crops (Koç, 2008; Paillisson & Marion, 2011), as well as morphological and structural characteristics of plants (Cooling, Ganf & Walker, 2001; Raulings et al., 2010). Consequently, we know relatively little of the community composition of RM and the primary factors governing it in lakeshore areas, especially in the subtropics.

The Yangtze River floodplain is one of the world’s largest, where numerous lakes were freely connected with the Yangtze’s main flow stem. However, due to intensive sluice construction and other human disturbances, these natural river-lake connections have been blocked for most lakes, leaving their water level fluctuations altered to various extents (Zhang, Liu & Wang, 2015), which probably change the community composition of the lakeshore RMs and probably influence the lakeshore plant metabolism and function. However, the influence of the water level fluctuations alter on the lakeshore RMs is rarely studied. To answer two main questions: (1) Are the lakeshore RMs located in the Yangtze River floodplain significantly different among different lakes, seasons, and sites? (2) What are the most important factors determining the microbial species composition and community structure of RMs in these subtropical lakeshore areas? In the present study, lakeshore RM was collected on a seasonal basis from three subtropical floodplain lakes located in the Yangtze River floodplain with different water level fluctuations.

## Material and Methods

### Sample collection

**Figure 1:**
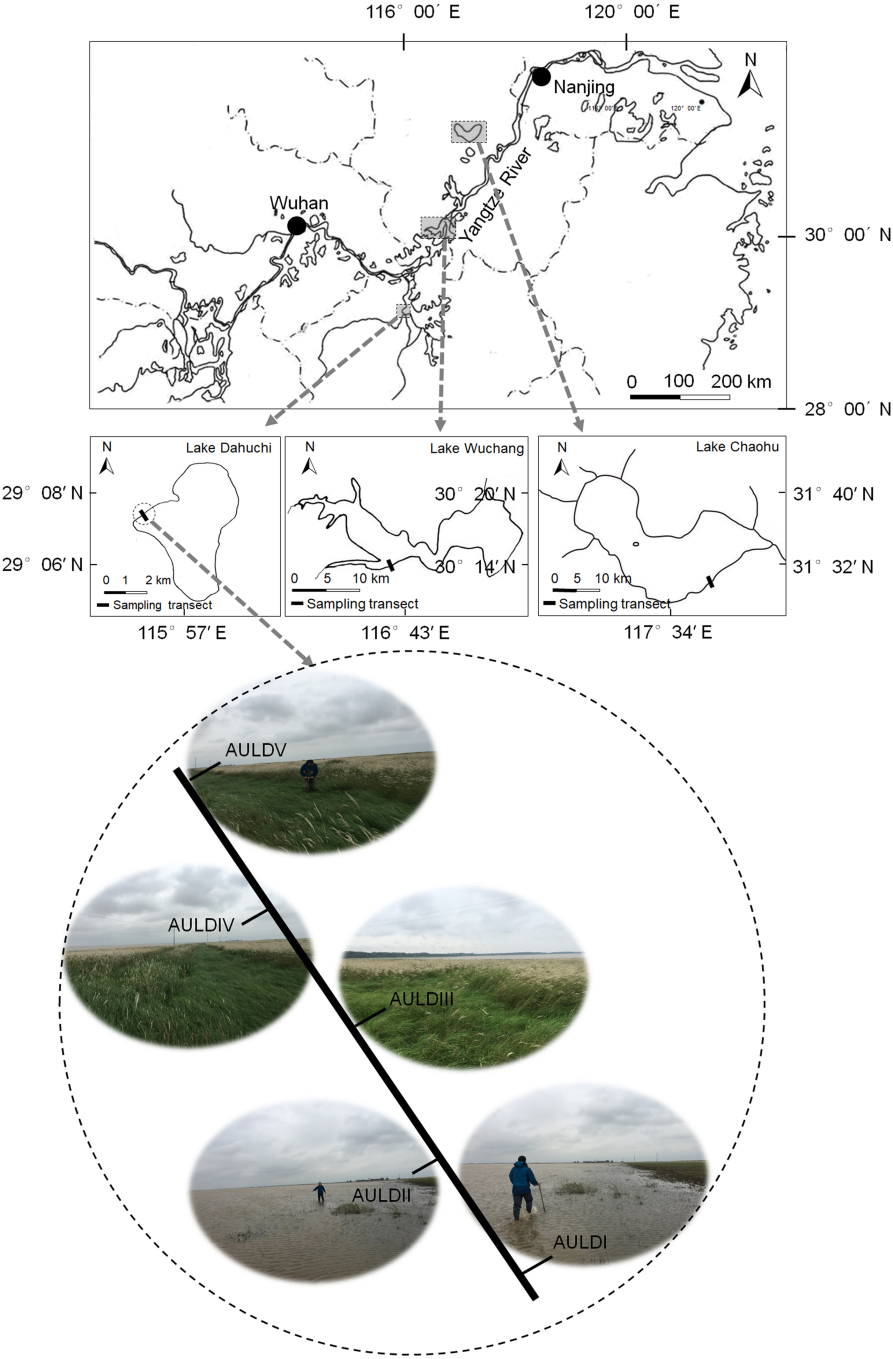
Location of the sampled three lakes and transects in this study. Along each transect, five sites numbered I, II, III, IV, and V were sequentially set perpendicular to the lakeshore from the mean annual lowest water level to its highest water level. The elevation differences were equal between any two adjacent sites in each transect. The average submerged depths of sampling sites were showed in Fig. S1(N). The sample group names were formed by combining sampling season, lake, and sampling site. AU, autumn; LD, Lake Dahuchi; hence AULDIII indicated the sample taken from the III site of Lake Dahuchi in autumn, 2016.

A detailed overview of the studied lakes and water level features of the sampling area was given in our previous report (Zhang et al., 2018). The RMs were collected from lakeshore areas of three lakes: Lake Chaohu (LC), Lake Wuchang (LW), and Lake Dahuchi (LD) in summer (August 28–31, 2016; SU), autumn (November 17–20, 2016; AU), winter (February 18–20, 2017; WI), and spring (May 13–15, 2017; SP). One transect free of any artificial disturbance was established at each lake (Fig. 1). Along each transect, five sites numbered I, II, III, IV, and V were sequentially set perpendicular to the lakeshore from the mean annual lowest water level to its highest water level (Fig. 1). The elevation differences were equal between any two adjacent sites in each transect. Three rhizosphere soil samples were randomly collected from each site using a portable root-soil core sampler (4.0 cm diameter with a 17 cm depth), and these samples mixed to form a composite sample. The latter was named according to the season, lake, and location of sampling: for instance, “SPLWIII” refers to a sample obtained from site III of Lake Wuchang in spring. A total of 58 samples were collected from the three lakes spanning four seasons; the samples SULDV and WILCV were not collected due to logistical limitations in the field.

### Measurement of physiochemical and hydrological variables

Rhizosphere soil pH and moisture were measured using the potentiometric method and the oven-drying method, respectively (Lu, 2000). Organic content (OC) was measured using the K_2_Cr_2_O_7_ titration method, while the total nitrogen (TN) and total phosphorus (TP) contents were measured respectively with the Kjeldahl method and molybdenum blue colorimetry (Lu, 2000). Daily average water level data from 2012–2016 was used to calculate the fluctuation range of the water level (FRWL), submerged duration (SD), and average submerged depth (ASD); the data were obtained from the Jiangxi Poyang Lake National Nature Reserve and relevant hydrological website (http://yc.wswj.net/ahsxx/LOL/public/public.html). The FRWL was calculated as the difference between the highest and lowest water level for a given lake within a calendar year. The SD was calculated as the sum of days the site was under water, and the ASD was calculated according to the relative elevation of each sampling site and daily hydrological data.

### DNA extraction and sequencing

Microbial DNA was extracted from samples using the SDS-based DNA extraction method, as done in earlier studies (Ni et al., 2010; Ni et al., 2012; Natarajan et al., 2016). The DNA integrity was checked by 1.0% agarose gel electrophoresis at 120 V for 30 min. The DNA concentration and purity were evaluated using a Nanodrop 2000 spectrophotometer (Thermo Scientific, USA) and then diluted to 1 ng/µl using sterile water. The V4 hypervariable region of prokaryotic 16S rRNA gene was amplified by using the prokaryotic specify primer set 515F and 806R with sample-specific barcode sequences, as done in earlier studies (Xiang et al., 2018). In brief, each 25-µl reaction mix contained 1 × PCR buffer, 0.25 U of *Taq* polymerase (Transgen Biotech, China), 0.2 mM of each dNTP, 1.0 µM of each primer, and 10 ng of microbial genomic DNA. The thermal cycling procedure was predenaturation at 94 °C for 10 min, followed by 30 cycles of 94 °C for 30 s, 56 °C for 30 s, and 72 °C for 30 s, with a final extension at 72 °C for 10 min. After this amplification, the PCR products were subjected to electrophoresis using a 2% agarose gel and quantified using a Nanodrop 2000 spectrophotometer (Thermo Scientific, USA). All amplicons were then pooled together with an equal molar amount from each sample (Huang et al., 2018) and purified using a DNA gel extraction kit (QIAGEN, Germany). Next, the pooled amplicons were sequenced using an Illumina HiSeq 2500 system at Beijing Novogene Technology Co., Ltd.

### Data processing and analysis

Raw data were merged with tags by using FLASH v1.2.7 (Magoč & Steven, 2011), and divided among the samples according to the barcode sequences using QIIME v1.9.0 (Caporaso et al., 2010). After removing the barcode and primer sequences, the reads of low-quality sequences were detected and removed in QIIME v1.9.0, as recently described by Huang et al. (2018). Next, any chimeras present were sought and filtered out by UCHIME software with the “Gold” database (http://drive5.com/uchime/uchime_download.html). The sequences were classified into operational taxonomic units (OTUs) by setting a threshold of 97% identified sequence by using UPARSE v7.0.1001 software (Edgar, 2013); the highest frequency sequence in each OTU was then selected as the representative sequence of the OTU.

Phylogenetic information for each OTU was annotated, according to the representative sequence, by using the Mothur pipeline-referenced SILVA SSUrRNA database (Quast et al., 2013). A phylogenetic tree of the OTUs was constructed in MUSCLE v3.8.31 (Edgar, 2004). Four alpha-diversity indexes—observed OTU counts, Chao1 index, Shannon index, and Good’s coverage—were calculated using QIIME v1.9.0. Rarefaction curve, and rank-abundance curve were drawn using R v2.15.3. The weighted UniFrac distances were calculated using QIIME v1.9.0.

All the bacterial sequences have been deposited in the NCBI SRA database under accession number SRP161734.

### Statistical analysis

Results for each variable are presented as the mean ± standard error for each group (Huang et al., 2018). The non-parametric Kruskal-Wallis rank sum test and post-hoc tests were used to identify significantly different taxa among different groups with STAMP software (Parks et al., 2014). Correspondence analysis (CA), canonical correspondence analysis (CCA), Non-metric multidimensional scaling (NMDS), and non-parametric multivariate analysis of variance (MANOVA) were conducted using the “vegan” package in the R platform. A heatmap profile was drawn using HemI software. Separate Wilcoxon tests were used to compare the *α*-diversity indexes between different groups, by using the agricolae package in R.

## Results

### Environmental variables and taxonomic composition of the RMs

**Figure 2:**
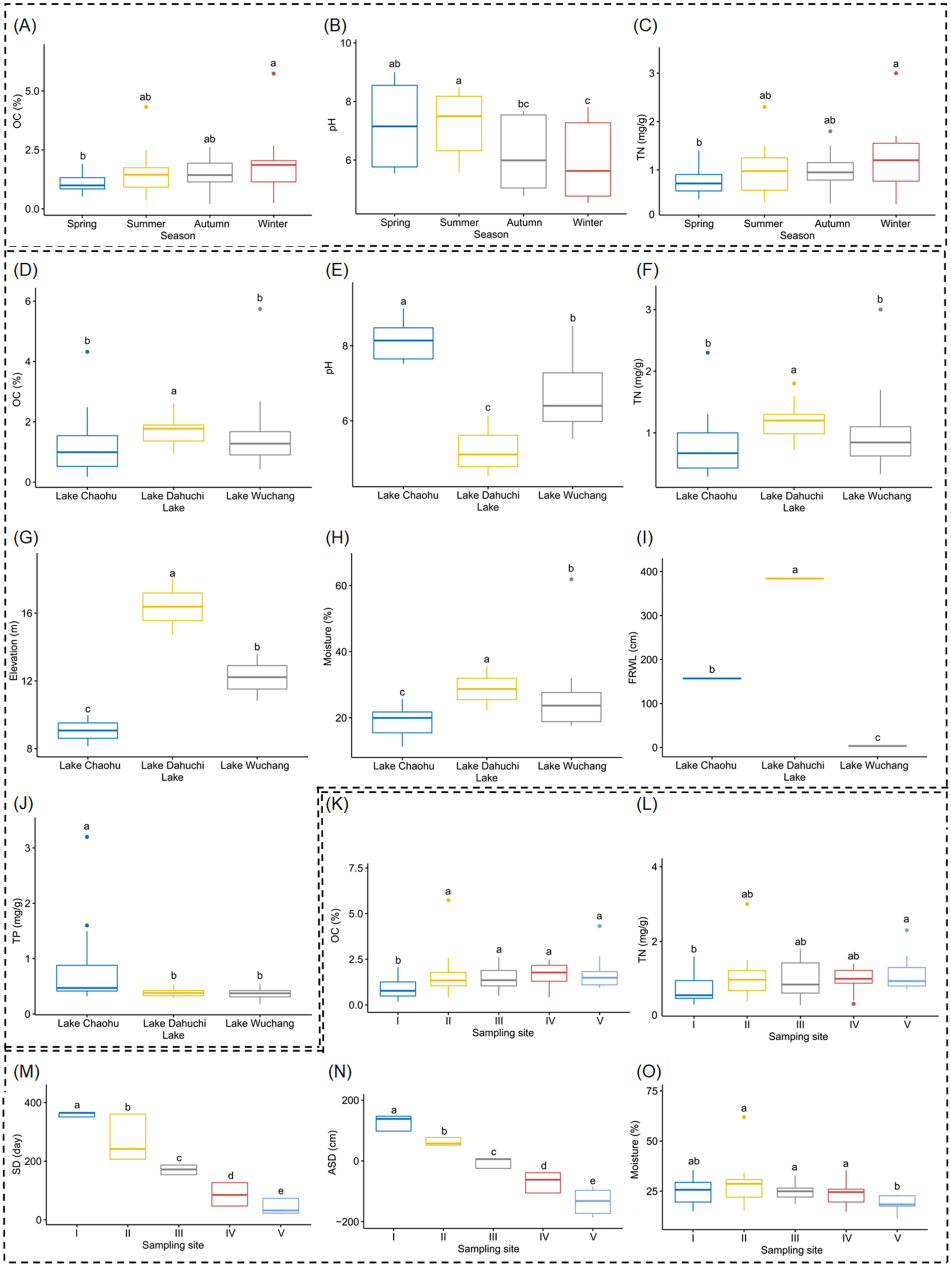
Boxplots (A-O) show the differences of environmental factors among seasons, among lakes, and among sampling sites. OC, organic content; TN, total nitrogen; FRWL, fluctuation range of the water level; TP, total phosphorus; SD, submerged duration; ASD, average submerged depth. Different lower case letters above the boxes indicate there were significant differences between the two groups (*p* < 0.05).

Both OC and TN differed significantly between spring and winter (Figs. 2A and 2C, and Table S1), as did soil pH, but it also was significantly different between summer and autumn, and likewise between summer and winter (Fig. 2B, and Table S1). Soil moisture, pH, elevation, and FRWL were significantly different among the three lakes. OC and TN were significantly different between LC and LD, and between LD and LW, while TP was significantly different between LC and LD, and between LC and LW (Figs. 2D–2J, and Table S1). The SD and ASD were significantly different among sampling sites; specifically, the OC of site I differed from all other sites; TN differed between sites I and V; moisture of site V differed from all other sites except I (Figs. 2K–2O, and Table S1).

**Figure 3:**
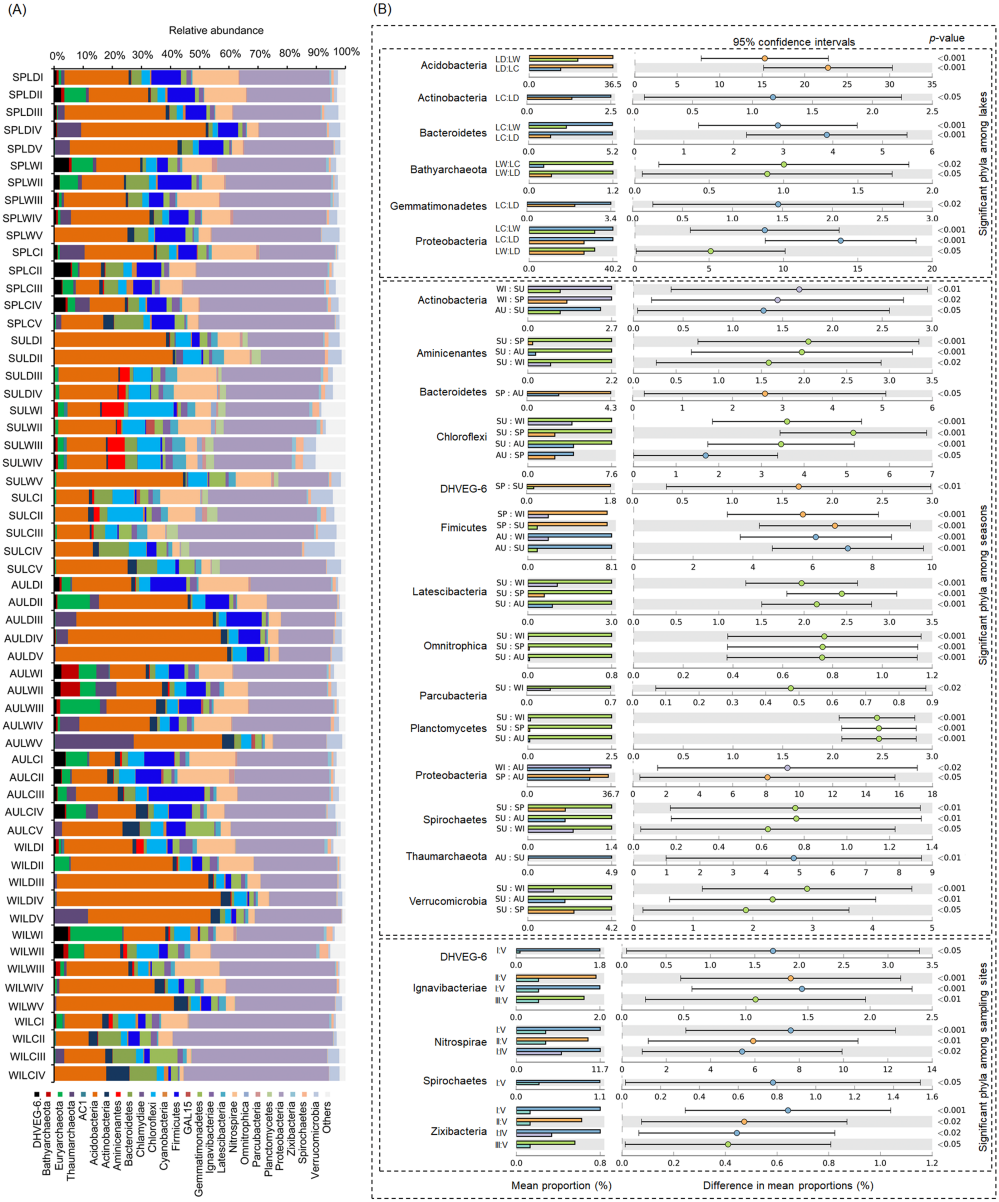
Dominant phyla (A) and significant dominant phyla (B) among different groups in the lakeshore rhizospheric microbiota. The sample group names were formed by combining sampling season, lake, and sampling site. SP, spring; SU, summer; AU, autumn; WI, winter; LC, Lake Chaohu; LW, Lake Wuchang; LD, Lake Dahuchi. Therefore, for example, SPLWIII indicated the sample taken from the III site of Lake Wuchang in spring, 2017.

After removing low quality sequences, a total of 2 488 433 (42 904.02 ± 1201.19 per sample) high-quality sequences were obtained for the 58 samples. To eliminate the influence of sequencing depth upon our results, all samples were randomly resampled to 24 038 sequences for further analysis, which was the lowest number of sequences per sample. Besides a few unclassified sequences (1.11 ± 0.14%), other sequences could be classified into 14 archaeal and 58 bacterial phyla. However, only four archaeal—i.e., Euryarchaeota (2.41 ± 0.47%), and Thaumarchaeota (2.31 ± 0.55%), DHVEG-6 (1.04 ± 0.19%), Bathyarchaeota (0.59 ± 0.15%)—and 21 bacterial phyla—Proteobacteria (32.64 ± 1.12%), Acidobacteria (23.79 ± 1.76%), Nitrospirae (8.16 ± 0.61%), Firmicutes (4.76 ± 0.54%), Chloroflexi (4.51 ± 0.33%), Bacteroidetes (2.98 ± 0.34%), Gemmatimonadetes (2.60 ± 0.22%), Verrucomicrobia (2.39 ± 0.27%), Actinobacteria (1.84 ± 0.19%), Ignavibacteriae (1.52 ± 0.11%), Latescibacteria (1.37 ± 0.15%), Spirochaetes (0.83 ± 0.09%), Aminicenantes (0.75 ± 0.20%), Planctomycetes (0.66 ± 0.15%), Zixibacteria (0.48 ± 0.05%), Parcubacteria (0.42 ± 0.06%), Cyanobacteria (0.41 ± 0.07%), Omnitrophica (0.20 ± 0.07%), GAL15 (0.17 ± 0.06%), AC1 (0.12 ± 0.03%), and Chlamydiae (0.08 ± 0.04%) —were found to be dominant, in that their relative abundances exceeded 1% in at least one of our samples (Fig. 3A). In Proteobacteria, Deltaproteobacteria was the most abundant class, followed by Betaproteobacteria, and Alphaproteobacteria (Table S2).

### Comparing the RMs among different lakes, seasons, and sites

Within these four archaeal and 21 bacterial phyla, the relative abundances of Acidobacteria, Actinobacteria, Bacteroidetes, Bathyarchaeota, Gemmatimonadetes, and Proteobacteria were significantly different among the three lakes. More than half of these dominant phyla also differed significantly among seasons in their relative abundance, while the relative abundance of DHVEG-6, Ignavibacteriae, Nitrospirae, Spirochaetes, and Zixibacteria were significant differences among the sampling sites (Fig. 3B).

Almost all the sequences (98.89 ± 0.14%) were classified into phyla, for a total of 21 361 OTUs detected. The rarefaction curve showed that most samples reached the platform, which implied that the sequences could represent the microbiota structures of the RMs (Fig. S1A). The rank-abundance curve showed that the OTUs in the microbiota were very uneven (Fig. S1B). Of the 21 361 OTUs, only 182 of them dominated the RMs (i.e., relative abundance >1% in at least one sample; Fig. S2), harboring 30.87 ± 1.53% of all the high-quality sequences, which were consistent with the result of the rank abundance curve (Fig. S1B). The microbiota first tended to cluster according to lakes, and cluster according to seasons within the same lake. However, no evidence was found to indicate the microbiota clustered according to sampling sites of the lakeshore areas (Fig. S2).

Spatially, there were 118 dominant OTUs for which we detected a significant difference in their relative abundance among the three lakes. The relative abundances of the dominant OTUs classified into *Nitrospira* cf. *moscoviensis* SBR1015, *Gaiella* sp., *Tenderia* sp., *Steroidobacter* sp., *Steroidobacter* sp. WWH78, *Methylomirabilis* sp., *Sphingomonas* sp., *Methylotenera* sp., *Sulfuricurvum* sp., *Methanosaeta* sp., *Nitrosoarchaeum* sp., *Clostridium beijerinckii*, *Thiobacillus* sp., *Eubacterium* sp., *Sulfurifustis* sp., family Gemmatimonadaceae, Gallionellaceae, Rhodospirillaceae, Nitrospiraceae, Desulfurellaceae, MIZ17, Sh765B-TzT-35, 0319-6A21, order Xanthomonadales, Holophagae, NB1-j, class Acidobacteria, and SAGMCG-1 were all significantly higher in LC than in the other two lakes. The relative abundances of the dominant OTUs classified into *Koribacter* sp., *Nitrotoga* sp., *Acidibacter* sp., *Solibacter* sp., *Terracidiphilus* sp., family Acidobacteriaceae, DA111, FW13, ASC21, Gallionellaceae, order Sva0485, Holophagae, class Acidobacteria, JG37-AG-4, and SAGMCG-1 were all significantly higher in LD than in the other two lakes. Finally, the relative abundances of the dominant OTUs classified into *Koribacter* sp., *Rhodanobacter* sp., *Geobacter* sp., *Methanoperedens* sp., *Nitrosotalea* sp., family Nitrospiraceae, Nitrosomonadaceae, Acetobacteraceae, Gemmatimonadaceae, order Sva0485, and MSBL5 were all significantly higher in LW than in the other two lakes.

Temporally, 112 of the 182 dominant OTUs detected showed significant differences in their relative abundance among the four seasons. In the summer, the dominant OTUs classified into *Clostridium beijerinckii*, *Thiobacillus* sp., family 0319-6A21, FW13, Acidobacteriaceae, Sh765B-TzT-35, order Holophagae, Sva0485, and Xanthomonadales were significantly increased, while those classified into *Nitrosoarchaeum* sp., and family Acidobacteriaceae significantly decreased. In autumn, the dominant OTUs classified into *Anaerostipes hadrus*, *Acetobacter pasteurianus*, *Acidibacter* sp., *Eubacterium* sp., *Faecalibacterium* sp., *Lactobacillus vini*, *Lactobacillus* sp., *Roseburia inulinivorans*, *Ruminococcus bicirculans*, *Ruminococcus* sp., *Subdoligranulum* sp., *Serratia marcescens*, *Methylomirabilis* sp., *Koribacter* sp., *Nitrosotalea* sp., *Methanoperedens* sp., family Nistrospiraceae, Gallionellaceae, and FW13 were significantly increased, while those classified into *Sideroxydans* sp., *Haliangium* sp., *Sulfuricurvum* sp., and family Nitrosomonadaceae, order NBi-j, class Acidobacteria, and ML635J-21 significantly decreased. In winter, the dominant OTUs classified into *Carnobacterium maltaromaticum*, *Telluria mixta*, *Enterobacter* sp., *Methylotenera* sp., *Solibacter* sp., family Methylococcaceae, Gemmatimonadaceae, order MSBL5, Holophagae, NB1-j, and Holophagae were significantly increased, while those classified into family Nitrospiraceae, MIZ17, 0319-6A21, order TRA3-20, and class Acidobacteria significantly decreased. Finally, in spring, the dominant OTUs classified into *Clostridium* sp. ND2, *Sideroxydans* sp., *Terracidiphilus* sp., *Geobacter* sp., *Arthromitus* sp., *Gallionella* sp., *Sulfuricurvum* sp., *Sphingomonas* sp., *Nitrotoga* sp., family Nitrospiraceae, NIZ17, Acidobacteriaceae, class Acidobacteria, ML635J-21, and SAGMCG-1 were significantly increased, while those classified into *Koribacter* sp., and class JG37-AG-4 significantly decreased.

Among sampling sites, we detected 43 dominant OTUs with significant differences across this elevation gradient. The relative abundances of the most significantly different dominant OTUs gradually changed with elevation: those classified into *Methanoperedens* sp., family ASC21, Gallionellaceae, Nitrospiraceae, order 43F-1404R, Sva0485, and MSBL5 were diminished from sampling site I through V, whereas those classified into *Solibacter* sp., *Sphingomonas* sp., *Terracidiphilus* sp., and family Acidobacteriaceae gradually increased.

More OTUs were detected from the lakeshore RMs in spring than in the other seasons (Kruskal-Wallis test, *χ*^2^ = 11.935, *p* = 0.008; Fig. S3A). The OTU counts at LC were more diverse than those occurring in the other two lakes (Kruskal-Wallis test, *χ*^2^ = 13.611, *p* = 0.001; Fig. S3B). Although there was a trend for the OTU counts to decline from sampling site I to V, no significant difference was detected among the sampling sites (Kruskal-Wallis test, *χ*^2^ = 4.878, *p* = 0.30; Fig. S3C). The Shannon indexes were not significantly different among the four seasons (Kruskal-Wallis test, *χ*^2^ = 7.396, *p* = 0.060; Fig. S3D). However, the Shannon indexes of the RMs at LC exceeded those of the other lakes (Kruskal-Wallis test, *χ*^2^ = 21.112, *p* < 0.001; Fig. S3E). No significant difference was detected among the five sampling sites in the Shannon index of the lakeshore RMs (Kruskal-Wallis test, *χ*^2^ = 2.619, *p* = 0.624; Fig. S3F). Chao 1 index of the lakeshore RMs in SP was significantly higher than other seasons (Fig. S3G), while the Goods’ coverage of the lakeshore RMs in SP was significantly lower than other seasons (Fig. S3J). No significant difference was detected among the lakes and sampling sites in the Chao1 index and Goods’ coverage (Figs. S3H, S3I, S3K, and S3L). Because the sampling sites were closely related to ASD, we also analyzed the correlation between the *α* diversity indexes and the ASD. Our results showed that except Shannon index significantly positively correlated with ASD (*F*-test, *F* = 4.35, *p* = 0.04; Fig. S4), OTU count, Chao1 index, and Goods’ coverage did not significantly correlate with ASD (*F*-test, *p* > 0.05; Fig. S4).

**Figure 4:**
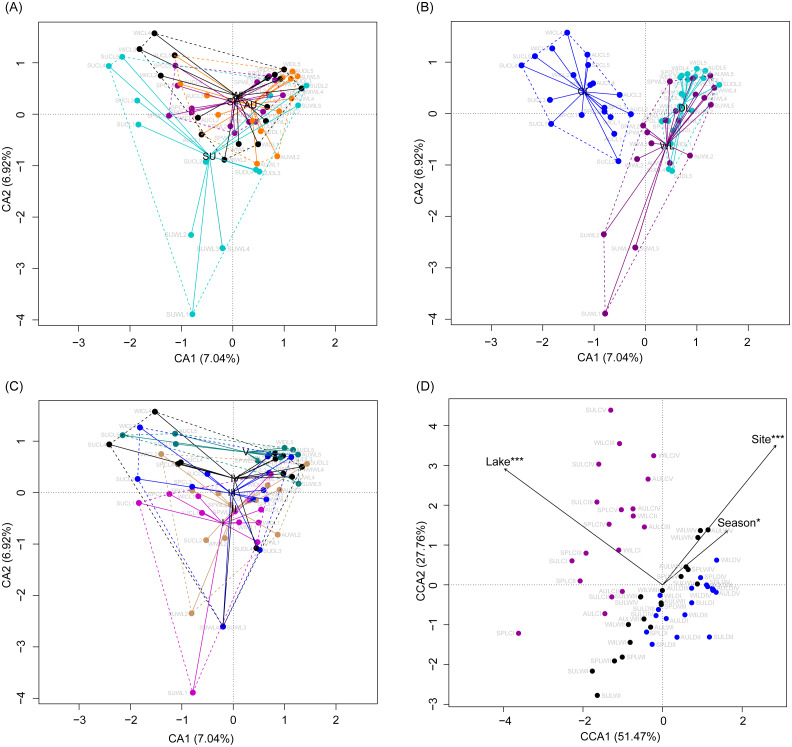
Correspondence analysis (A, B, and C) and canonical correspondence analysis (D) profiles of samples based on OTUs in lakeshore rhizospheric microbiota. Different colors indicate different seasons (A), lakes (B), and sampling sites (C). The sample group names were formed by combining sampling season, lake, and sampling site. SP, spring; SU, summer; AU, autumn; WI, winter; LC, Lake Chaohu; LW, Lake Wuchang; LD, Lake Dahuchi. Therefore, for example, SPLWIII indicated the sample taken from the III site of Lake Wuchang in spring, 2017.

Although the CA based on all OTUs did not absolutely distinguish the RMs from different lakes, seasons, or sampling sites (Figs. 4A–4C), the CCA with Monte Carlo testing and MANOVA revealed significant differences in the RMs among lakes (MANOVA, *F* = 5.99, *p* < 0.01), seasons (MANOVA, *F* = 3.51, *p* < 0.01), and sampling sites (MANOVA, *F* = 4.03, *p* < 0.01) (Fig. 4D). NMDS result also showed that the RMs had trends to distinguish according to lakes and sampling sites (Fig. S5).

**Figure 5:**
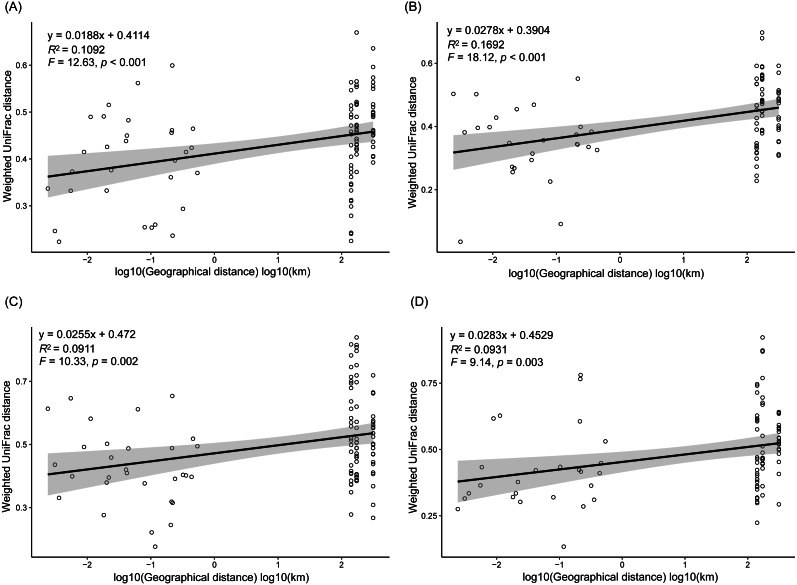
Correlation of the weighted UniFrac distances between the sampling sites to the geographic distances in spring (A), summer (B), autumn (C), and winter (D).

As dispersal limitation caused by geographic distance is one of the major mechanisms that maintain *β*-diversity of microbial communities (Eisenlord, Zak & Upchurch, 2012; Ni et al., 2014; Cao et al., 2016; Shirani & Hellweger, 2017), we analyzed the variation of the weighted UniFrac distances between the sampling sites with the geographic distances. Our results showed that the weighted UniFrac distances significantly increased with the increases of the geographic distances (Fig. 5). However, the *R*^2^ of the regression equations were very low (<0.20; Fig. 5).

### Influence of environmental factors on the taxonomic composition of RMs

**Figure 6:**
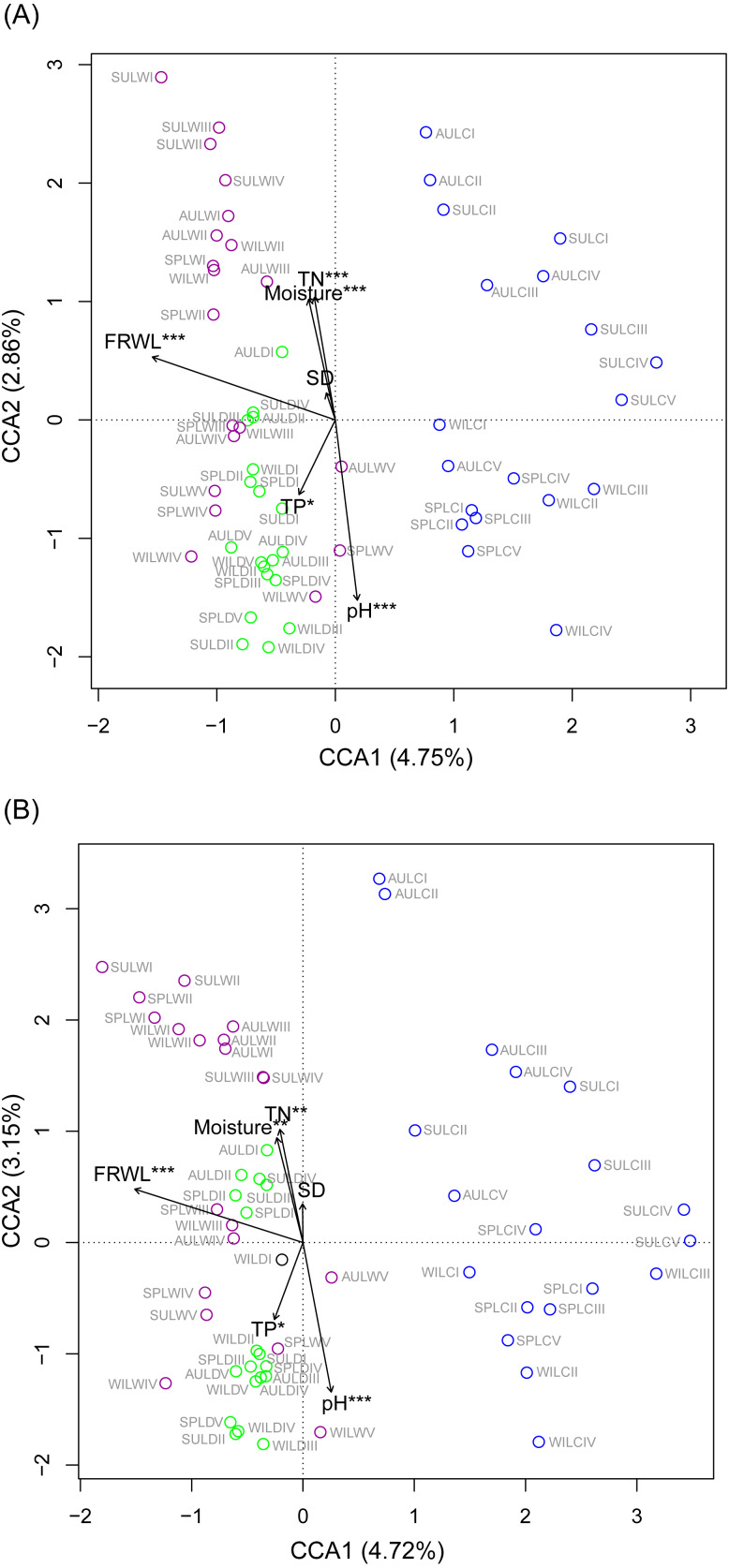
Canonical correspondence analysis profiles showing the influence of various environmental factors on rhizospheric microbiota (A) and dominant microbiota (B). Different colors indicate different sampling lakes. The sample group names were formed by combining sampling season, lake, and sampling site. SP, spring; SU, summer; AU, autumn; WI, winter; LC, Lake Chaohu; LW, Lake Wuchang; LD, Lake Dahuchi. Therefore, for example, SPLWIII indicated the sample taken from the III site of Lake Wuchang in spring, 2017. TN, total nitrogen; TP, total phosphorus; SD, submerged duration; FRWL, fluctuation range of water level.

CCA was used to analyze which environmental factors best explained the microbiota structure in lakeshore rhizospheres. Since SD and ASD, pH and elevation, and OC and TN were significantly correlated with each other (the absolute value of Pearson correlation *R*-values >0.9 and *p*-values < 0.001; Fig. S6), the elevation, OC, and ASD variables were deleted before conducting the CCA. When performed with the Monte Carlo test, it showed that FRWL and pH (or elevation) were the most important factors, followed by TN (or OC), moisture and TP, for significantly influencing the lakeshore RMs (Fig. 6A) and their dominant microbiota (Fig. 6B).

To analyze which dominant OTUs were influenced by hydrological and soil physiochemical variables, linear regressions were used. The FRWL was a positive predictor of the dominant OTUs in the genus Candidatus *Koribacter*, genus Candidatus *Solibacter*, family Acidobacteriaceae, and class Acidobacteria, but it was negatively related to the dominant OTUs in the genus Candidatus *Methanoperedens* and the phylum Bathyarchaeota (Table S3). A greater soil pH negatively affected the dominant OTUs in the genus Candidatus *Koribacter*, genus Candidatus *Solibacter*, genus *Sphingomonas*, genus RB41, family Rhodospirillaceae, family Acidobacteriaceae, family FW13, order Sva0485, and class Acidobacteria, but positively influenced those OTUs in the genus *Geobacter*, family Nitrospiraceae, order 43F-1404R, order Sva0485, and phylum Bathyarchaeota. Both soil TN and TP largely had a positive influence on the dominant OTUs in the family 0319-6A21 and family Rhodospirillaceae, respectively (Table S3).

## Discussion

Rhizospheric microbiota (RM) not only play significant roles in plant growth, nutrition, and health (Mendes et al., 2011; Philippot et al., 2013; Panke-Buisse et al., 2015; Gill et al., 2016), but they can directly affect a wide range of ecosystem processes (Fierer & Jackson, 2006). Hence, maintaining rhizosphere microbial diversity was necessary to persist the ecological functions of RM (Schimel, Bennett & Fierer, 2005; Standing et al., 2005). In the present study, we found that the compositions of RM were different among lakes, seasons, and elevation sites in subtropical lakeshore areas located in the Yangtze River floodplain (Fig. 4), with hydrological and soil physiochemical variables, such as the FRWL, rhizosphere soil pH (or elevation as it significantly correlated with pH; Fig. S6), TN (or OC as it significantly correlated with TN; Fig. S6), and TP, being the major factors driving the observed bacterial changes over space and time (Fig. 6).

Hydrological conditions are usually taken into consideration when studying plants in lake habitats (Van Geest et al., 2005; Raulings et al., 2010). In the Yangtze River floodplain lakes, Zhang et al. (2018) concluded the FRWL was the most important factor for determining the distribution of lakeshore plants, followed by relative elevation and SD. Further, hydrological factors were also strongly correlated with the architectural and morphological traits of plant roots in lakeshore areas of Yangtze floodplain lakes. Taken together, this demonstrates hydrology’s importance for affecting the structure and function of above-ground and below-ground tissues of lakeshore plants.

In the measured physiochemical variables in our study, FRWL was a factor that significantly influenced lakeshore RM, likely because FRWL not only can affect RM directly but it also influences the lakeshore’s plant composition, root development, and soil physiochemical variables (such as the level of oxygen in soil). Several studies have indicated that plant community diversity and the genotypes of individual plants can influence the composition of their associated RM communities in non-cultivated ecosystems (Whitham et al., 2006; Schweitzer et al., 2008; Philippot et al., 2013). For our three lakes, their lakeshore plant communities are easily classified into distinguishable layers, with the distribution of plants in each layer relatively uniform in response to different hydrological conditions, and the spatial configuration of their root systems is distinct among the three lakes (Zhang et al., 2018). Therefore, the indirect effects caused by hydrology contribute importantly to the RM differences found among the lakes, seasons, and sites in our study.

Soil pH was another factor significantly influencing the RM in this study. The stress of residing in suboptimal pH environments is known to impact the diversity and composition of microbial communities in a range of terrestrial and aquatic environments (Hörnström, 2002; Bååth & Anderson, 2003). Fierer & Jackson (2006) even found that the diversity of soil bacterial communities was unrelated to site temperature, latitude, and other environmental variables, but was strongly affected by soil pH, with bacterial diversity highest in neutral soils but lower in acidic soils. Therefore, significantly different pH conditions across the three lakes likely contributed in a large way to the differing RM community compositional structure that we found (Fig. 2E).

TN was significantly different between lakes, and between seasons (Fig. 2), and also significantly influenced the structure of RMs. Many significantly different OTUs, such as *Nitrospira* sp. (Hovanec et al., 1998; Lücker et al., 2010), *Nitrosotalea* (Restrepo-Ortiz et al., 2018), and *Nitrotoga* (Lücker et al., 2015), participate in nitrogen or sulfur cycle in freshwater habitats. Those microorganisms may be more sensitive to TN, which could explain the differences in their OTUs between lakes and seasons. That microbiota compositional differences between seasons can continually persist has been reported (Yan et al., 2017). The significantly different OTUs between seasons—such as *Ruminococcus* sp. (Ze et al., 2012), *Faecalibacterium* sp. (Benus et al., 2010), and *Clostridium* sp. (Loo et al., 2005; Kim, Jeong & Chun, 2007)—are commonly found in human gut and aquatic sediment, where they participate in polysaccharide metabolism. Their respective polysaccharide metabolism capacity probably plays a critical role in the carbon cycle of lakeshores and is significantly influenced by seasons.

The compositions of the RMs in different seasons over a 1-yr period also changed considerably (Fig. 4D). Since plant developmental stage, climate and other environmental factors fluctuate seasonally, the changes in RMs across seasons were reasonably expected (Williams et al., 2013). However, seasonality probably did not directly change the RMs, but rather it indirectly changed them via altered soil physiochemical variables: TN, OC, and pH were significantly different between seasons (Fig. 2) and they significantly influenced the RMs (Fig. 6). However, considering these variables together explained just a very small portion of RM diversity (Fig. 6), we therefore suggest many unmeasured variables such as soil temperature probably also significantly influenced the RM communities, as shown in other work (Xue et al., 2015).

It is also worthwhile to compare broadly the phylum composition of lake sediment microbiota, which is typically dominated by Firmicutes, Proteobacteria, Bacteroidetes, Actinobacteria, and Chiloroflexi (Krett & Palatinszky, 2009; Paul et al., 2016; Korenblum, Jiménez & Elsas, 2016). The phyla dominating the RMs in our subtropical lakeshore areas are similar to those occurring in terrestrial environments (Uroz et al., 2010; Mendes et al., 2011; Peiffer et al., 2013), despite the RMs we examined being dominated by Firmicutes, Proteobacteria, and Actinobacteira (Fig. 3). This result suggests that the rhizosphere ecosystem could specifically enrich the microbiota originating from soil environments, not unlike that found elsewhere (Berg & Smalla, 2009; Philippot et al., 2013).

## Conclusions

In conclusion, by investigating the composition of RMs in three lakes over four seasons and among five sampling sites, we found that different microbial phyla exhibited differential responses to changes in season, lake, and habitat. The Acidobacteria, Actinobacteria, Bacteroidetes, Bathyarchaeota, Gemmatimonadetes, and Proteobacteria all differed considerably among the three lakes, with more than half of these dominant phyla showing marked changes in abundance between seasons, while DHVEG-6, Ignavibacteriae, Nitrospirae, Spirochaetes, and Zixibacteria varied substantially across the sampling sites. The fluctuation range of water level and pH were the most important factors significantly influencing the rhizospheric microbial communities and their dominant microbiota, followed by TN, moisture, and TP in soil. Additionally, the weighted UniFrac distances between sampling sites significantly increased with the increases of the geographic distances.

##  Supplemental Information

10.7717/peerj.10078/supp-1 Table S1 Physiochemical and hydrological variables of sampling sites OC, organic content; TN, total nitrogen; TP, total phosphorus; FRWL, fluctuation range of the water level; SD, submerged duration; ASD, average submerged depth.

10.7717/peerj.10078/supp-2 Table S2 Relative abundances of classes of Proteobacteria in rhizospheric microbiota

10.7717/peerj.10078/supp-3 Table S3 Relationships between hydrological and soil physiochemical variables and relative abundances of dominant OTUs

10.7717/peerj.10078/supp-4 Figure S1 Rarefaction curve (A) and rank-abundance curve (B) of the rhizospheric microbiota Along each transect, five sites numbered I, II, III, IV, and V were sequentially set perpendicular to the lakeshore from the mean annual lowest water level to its highest water level. The elevation differences were equal between any two adjacent sites in each transect. The sample group names were formed by combining sampling season, lake, and sampling site. AU, autumn; LD, Lake Dahuchi; hence AULDIII indicated the sample taken from the III site of Lake Dahuchi in autumn, 2016.

10.7717/peerj.10078/supp-5 Figure S2 Heatmap profile of dominant OTUs in lakeshore rhizospheric microbiota. The sample group names were formed by combining sampling season, lake, and sampling site SP, spring; SU, summer; AU, autumn; WI, winter; LC, Lake Chaohu; LW, Lake Wuchang; LD, Lake Dahuchi. Therefore, for example, SPLWIII indicated the sample taken from the III site of Lake Wuchang in spring, 2017.

10.7717/peerj.10078/supp-6 Figure S3 Differences of alpha diversity indexes of rhizospheric microbiota between seasons, lakes, and sampling sites

10.7717/peerj.10078/supp-7 Figure S4 The regression of alpha-diversity indexes of rhizospheric microbiota to the average submerged depth (ASD)

10.7717/peerj.10078/supp-8 Figure S5 Non-metric multidimensional scaling profiles emphasized by sampling lakes (A) and sampling sites (B) The arrows indicate the trend from sampling site I to sampling site V. The sample group names were formed by combining sampling season, lake, and sampling site. AU, autumn; LD, Lake Dahuchi; hence AULDIII indicated the sample taken from the III site of Lake Dahuchi in autumn, 2016.

10.7717/peerj.10078/supp-9 Figure S6 Pearson correlation matrix of environmental factors The values in the upper right of the picture were the Pearson correlation coefficients between the environmental factors corresponding to the row and column of the values. Red and blue values indicate positive and negative correlations, respectively. The scatter plots and line chart in each panel in the lower left of the picture showed the points on the coordinates formed by the values of the environmental factors corresponding to the row and column of the panel at each sampling point, and the correlation curve of the two environmental factors fitted according to these points. “Lake” referred the sampling lakes, i.e. Lake Chaohu, Lake Wuchang, and Lake Dahuchi; “Season” referred the sampling seasons, i.e. summer, autumn, winter, and spring. TN, total nitrogen; TP, total phosphorus; SD, submerged duration; FRWL, fluctuation range of water level; ASD, average submerged depth; OC, organic content; SS, sampling site. *, *p* < 0.05; **, *p* < 0.01; ***, *p* < 0.001.

## Additional Information and Declarations

### Field Study Permissions

The following information was supplied relating to field study approvals (i.e., approving body and any reference numbers): Only rhizospheric microbiota of lakeshore plants were collected in this study, and the field work did not involve any cherished animals and plants, so no license agreement is needed in this study.

### Data Availability

The following information was supplied regarding data availability: Sequencing data are available at the NCBI Sequence Read Archive database: SRP161734.
