# Supplementary material for: Hydrological and soil physiochemical variables determine the rhizospheric microbiota in subtropical lakeshore areas
Source: PeerJ. 2020 Sep 29;8:e10078. doi: 10.7717/peerj.10078 (PMC7531358; doi:10.7717/peerj.10078)
Supplement: Table S1 — OC, organic content; TN, total nitrogen; TP, total phosphorus; FRWL, fluctuation range of the water level; SD, submerged duration; ASD, average submerged depth. [file peerj-08-10078-s001.docx]

**Table S1.** Physiochemical and hydrological variables of sampling sites. OC, organic content; TN, total nitrogen; TP, total phosphorus; FRWL, fluctuation range of the water level; SD, submerged duration; ASD, average submerged depth.

| Sample ID | pH | moisture | OC | TN | TP | Elevation | FRWL | SD | ASD |
| --- | --- | --- | --- | --- | --- | --- | --- | --- | --- |
| SULCI | 8.19 | 16.20965 | 0.363 | 0.44 | 0.76 | 8.15 | 157 | 365 | 98.1 |
| SULCII | 8.14 | 22.35441 | 0.981 | 0.67 | 0.32 | 8.61 | 157 | 361 | 52.1 |
| SULCIII | 8.44 | 25.67216 | 1.65 | 1 | 0.33 | 9.07 | 157 | 187 | 6.1 |
| SULCIV | 8.47 | 23.83952 | 2.48 | 1.3 | 1 | 9.52 | 157 | 47 | -38.9 |
| SULCV | 8.21 | 17.57869 | 4.32 | 2.3 | 3.2 | 9.98 | 157 | 20 | -84.9 |
| SULDI | 5.89 | 24.44591 | 1.37 | 1 | 0.3 | 14.75 | 384 | 351 | 138.5 |
| SULDII | 5.57 | 30.43084 | 1.77 | 1.3 | 0.38 | 15.56 | 384 | 207 | 57.5 |
| SULDIII | 5.63 | 32.90993 | 2.34 | 1.5 | 0.33 | 16.38 | 384 | 154 | -24.5 |
| SULDIV | 6.14 | 35.41511 | 1.52 | 1.1 | 0.4 | 17.19 | 384 | 85 | -105.5 |
| SULWI | 7.41 | 29.02208 | 0.763 | 0.42 | 0.3 | 10.82 | 3.45 | 365 | 147.1 |
| SULWII | 7.59 | 28.71553 | 1.32 | 0.95 | 0.42 | 11.52 | 3.45 | 242 | 77.1 |
| SULWIII | 7.6 | 23.14425 | 0.898 | 0.55 | 0.38 | 12.22 | 3.45 | 172 | 7.1 |
| SULWIV | 7.39 | 18.08912 | 0.422 | 0.33 | 0.18 | 12.91 | 3.45 | 127 | -61.9 |
| SULWV | 6.86 | 23.07057 | 1.66 | 0.83 | 0.35 | 13.61 | 3.45 | 73 | -131.9 |
| AULCI | 7.52 | 15.48106 | 0.186 | 0.31 | 0.56 | 8.15 | 157 | 365 | 98.1 |
| AULCII | 7.58 | 15.43239 | 0.407 | 0.4 | 0.48 | 8.61 | 157 | 361 | 52.1 |
| AULCIII | 7.58 | 22.83994 | 1.17 | 0.69 | 0.41 | 9.07 | 157 | 187 | 6.1 |
| AULCIV | 7.56 | 25.29138 | 2.2 | 1.3 | 0.41 | 9.52 | 157 | 47 | -38.9 |
| AULCV | 7.67 | 12.50539 | 1.43 | 1.3 | 1.6 | 9.98 | 157 | 20 | -84.9 |
| AULDI | 5.1 | 30.52632 | 1.11 | 0.95 | 0.41 | 14.75 | 384 | 351 | 138.5 |
| AULDII | 4.99 | 29.12844 | 1.36 | 1 | 0.3 | 15.56 | 384 | 207 | 57.5 |
| AULDIII | 4.77 | 32.46506 | 2.61 | 1.8 | 0.43 | 16.38 | 384 | 154 | -24.5 |
| AULDIV | 4.88 | 27.65685 | 2.16 | 0.91 | 0.44 | 17.19 | 384 | 85 | -105.5 |
| AULDV | 4.78 | 23.07076 | 1.45 | 0.97 | 0.34 | 18 | 384 | 32 | -186.5 |
| AULWI | 6.36 | 27.02559 | 1.24 | 0.89 | 0.34 | 10.82 | 3.45 | 365 | 147.1 |
| AULWII | 5.89 | 31.89021 | 2.57 | 1.5 | 0.31 | 11.52 | 3.45 | 242 | 77.1 |
| AULWIII | 5.99 | 25.86256 | 1.09 | 0.69 | 0.28 | 12.22 | 3.45 | 172 | 7.1 |
| AULWIV | 5.96 | 27.51905 | 1.71 | 1 | 0.28 | 12.91 | 3.45 | 127 | -61.9 |
| AULWV | 6.04 | 18.01914 | 1.53 | 0.91 | 0.39 | 13.61 | 3.45 | 73 | -131.9 |
| WILCI | 7.74 | 14.99375 | 0.25 | 0.49 | 0.45 | 8.15 | 157 | 365 | 98.1 |
| WILCII | 7.63 | 19.97301 | 1.07 | 0.69 | 0.42 | 8.61 | 157 | 361 | 52.1 |
| WILCIII | 7.81 | 19.35096 | 0.512 | 0.29 | 0.35 | 9.07 | 157 | 187 | 6.1 |
| WILCIV | 7.7 | 14.81136 | 1.95 | 1 | 1.5 | 9.52 | 157 | 47 | -38.9 |
| WILDI | 4.69 | 35.48718 | 2.07 | 1.6 | 0.43 | 14.75 | 384 | 351 | 138.5 |
| WILDII | 4.58 | 28.72377 | 1.74 | 1.2 | 0.33 | 15.56 | 384 | 207 | 57.5 |
| WILDIII | 4.59 | 25.70573 | 1.89 | 1.2 | 0.31 | 16.38 | 384 | 154 | -24.5 |
| WILDIV | 4.53 | 25.4902 | 1.84 | 1.2 | 0.38 | 17.19 | 384 | 85 | -105.5 |
| WILDV | 5.01 | 22.34186 | 1.88 | 1.3 | 0.46 | 18 | 384 | 32 | -186.5 |
| WILWI | 6.2 | 21.54097 | 0.729 | 0.54 | 0.38 | 10.82 | 3.45 | 365 | 147.1 |
| WILWII | 5.52 | 61.91932 | 5.74 | 3 | 0.5 | 11.52 | 3.45 | 242 | 77.1 |
| WILWIII | 6.01 | 24.26733 | 1.36 | 1.7 | 0.31 | 12.22 | 3.45 | 172 | 7.1 |
| WILWIV | 5.55 | 21.99497 | 2.33 | 1.4 | 0.43 | 12.91 | 3.45 | 127 | -61.9 |
| WILWV | 5.71 | 17.64923 | 2.68 | 1.6 | 0.49 | 13.61 | 3.45 | 73 | -131.9 |
| SPLCI | 8.49 | 20.74199 | 0.533 | 0.53 | 0.45 | 8.15 | 157 | 365 | 98.1 |
| SPLCII | 8.57 | 21.17235 | 0.668 | 0.39 | 0.47 | 8.61 | 157 | 361 | 52.1 |
| SPLCIII | 8.72 | 20 | 1.35 | 0.55 | 0.55 | 9.07 | 157 | 187 | 6.1 |
| SPLCIV | 8.64 | 20.15288 | 0.633 | 0.42 | 0.44 | 9.52 | 157 | 47 | -38.9 |
| SPLCV | 9 | 11.27321 | 0.993 | 0.8 | 1 | 9.98 | 157 | 20 | -84.9 |
| SPLDI | 5.6 | 31.51125 | 1.32 | 0.95 | 0.53 | 14.75 | 384 | 351 | 138.5 |
| SPLDII | 5.55 | 34.1995 | 1.82 | 1.2 | 0.42 | 15.56 | 384 | 207 | 57.5 |
| SPLDIII | 5.72 | 28.64949 | 1.9 | 1.4 | 0.32 | 16.38 | 384 | 154 | -24.5 |
| SPLDIV | 5.55 | 25.52806 | 1.32 | 1 | 0.34 | 17.19 | 384 | 85 | -105.5 |
| SPLDV | 5.81 | 22.89474 | 0.946 | 0.72 | 0.33 | 18 | 384 | 32 | -186.5 |
| SPLWI | 8.53 | 28.08146 | 0.789 | 0.58 | 0.54 | 10.82 | 3.45 | 365 | 147.1 |
| SPLWII | 6.44 | 26.81018 | 1.31 | 0.86 | 0.39 | 11.52 | 3.45 | 242 | 77.1 |
| SPLWIII | 7.15 | 18.7672 | 0.902 | 0.64 | 0.37 | 12.22 | 3.45 | 172 | 7.1 |
| SPLWIV | 6.74 | 18.11765 | 1.23 | 0.8 | 0.36 | 12.91 | 3.45 | 127 | -61.9 |
| SPLWV | 7.24 | 18.86429 | 0.936 | 0.72 | 0.53 | 13.61 | 3.45 | 73 | -131.9 |
