# Supplementary material for: Hydrological and soil physiochemical variables determine the rhizospheric microbiota in subtropical lakeshore areas
Source: PeerJ. 2020 Sep 29;8:e10078. doi: 10.7717/peerj.10078 (PMC7531358; doi:10.7717/peerj.10078)
Supplement: Table S2 [file peerj-08-10078-s002.docx]

Table S2. Relative abundances of classes of Proteobacteria in rhizospheric microbiota.

| Sample ID | Alphaproteobacteria | Betaproteobacteria | Deltaproteobacteria | Epsilonproteobacteria | FGL7S | Gammaproteobacteria | JTB23 | SPOTSOCT00m83 | Unidentified_Proteobacteria |
| --- | --- | --- | --- | --- | --- | --- | --- | --- | --- |
| SPLDI | 0.0332 | 0.0428 | 0.2085 | 0.0003 | 0.0000 | 0.0230 | 0.0000 | 0.0000 | 0.0002 |
| SPLDII | 0.0263 | 0.0511 | 0.1858 | 0.0049 | 0.0000 | 0.0171 | 0.0000 | 0.0000 | 0.0006 |
| SPLDIII | 0.0841 | 0.0708 | 0.1096 | 0.0079 | 0.0000 | 0.0243 | 0.0000 | 0.0000 | 0.0029 |
| SPLDIV | 0.0628 | 0.0484 | 0.0915 | 0.0010 | 0.0000 | 0.0268 | 0.0000 | 0.0000 | 0.0000 |
| SPLDV | 0.1177 | 0.0530 | 0.0851 | 0.0004 | 0.0000 | 0.0491 | 0.0000 | 0.0000 | 0.0000 |
| SPLWI | 0.0358 | 0.0769 | 0.2433 | 0.0002 | 0.0000 | 0.0170 | 0.0000 | 0.0000 | 0.0000 |
| SPLWII | 0.0279 | 0.1138 | 0.2078 | 0.0000 | 0.0000 | 0.0121 | 0.0000 | 0.0000 | 0.0000 |
| SPLWIII | 0.0445 | 0.1176 | 0.1953 | 0.0007 | 0.0000 | 0.0198 | 0.0000 | 0.0000 | 0.0000 |
| SPLWIV | 0.0723 | 0.0963 | 0.1301 | 0.0008 | 0.0000 | 0.0187 | 0.0000 | 0.0000 | 0.0000 |
| SPLWV | 0.0799 | 0.1177 | 0.1420 | 0.0003 | 0.0000 | 0.0326 | 0.0000 | 0.0000 | 0.0000 |
| SPLCI | 0.0366 | 0.0607 | 0.1192 | 0.0040 | 0.0000 | 0.0349 | 0.0000 | 0.0000 | 0.0029 |
| SPLCII | 0.0394 | 0.2038 | 0.1275 | 0.0397 | 0.0000 | 0.0338 | 0.0000 | 0.0002 | 0.0044 |
| SPLCIII | 0.0665 | 0.2192 | 0.1247 | 0.0191 | 0.0000 | 0.0409 | 0.0000 | 0.0001 | 0.0121 |
| SPLCIV | 0.0987 | 0.1498 | 0.1246 | 0.0050 | 0.0000 | 0.0529 | 0.0000 | 0.0002 | 0.0027 |
| SPLCV | 0.1630 | 0.1263 | 0.0928 | 0.0057 | 0.0000 | 0.0728 | 0.0000 | 0.0000 | 0.0035 |
| SULDI | 0.0619 | 0.0567 | 0.1173 | 0.0002 | 0.0000 | 0.0232 | 0.0000 | 0.0000 | 0.0000 |
| SULDII | 0.0675 | 0.0314 | 0.0924 | 0.0000 | 0.0000 | 0.0284 | 0.0000 | 0.0000 | 0.0000 |
| SULDIII | 0.0422 | 0.0956 | 0.1706 | 0.0002 | 0.0000 | 0.0314 | 0.0000 | 0.0000 | 0.0000 |
| SULDIV | 0.0327 | 0.0821 | 0.1753 | 0.0000 | 0.0000 | 0.0283 | 0.0000 | 0.0000 | 0.0000 |
| SULWI | 0.0040 | 0.0138 | 0.2587 | 0.0000 | 0.0000 | 0.0106 | 0.0000 | 0.0000 | 0.0000 |
| SULWII | 0.0171 | 0.0825 | 0.2003 | 0.0002 | 0.0000 | 0.0182 | 0.0000 | 0.0000 | 0.0000 |
| SULWIII | 0.0147 | 0.0659 | 0.1621 | 0.0010 | 0.0000 | 0.0239 | 0.0000 | 0.0000 | 0.0000 |
| SULWIV | 0.0146 | 0.0643 | 0.1624 | 0.0012 | 0.0000 | 0.0224 | 0.0000 | 0.0000 | 0.0000 |
| SULWV | 0.0439 | 0.0459 | 0.0758 | 0.0000 | 0.0000 | 0.0055 | 0.0000 | 0.0000 | 0.0000 |
| SULCI | 0.0281 | 0.1060 | 0.1407 | 0.0002 | 0.0000 | 0.0622 | 0.0000 | 0.0002 | 0.0000 |
| SULCII | 0.0228 | 0.1327 | 0.1435 | 0.0050 | 0.0000 | 0.0339 | 0.0000 | 0.0000 | 0.0039 |
| SULCIII | 0.0617 | 0.1185 | 0.1903 | 0.0001 | 0.0000 | 0.0959 | 0.0000 | 0.0002 | 0.0002 |
| SULCIV | 0.0566 | 0.1312 | 0.1136 | 0.0011 | 0.0000 | 0.0807 | 0.0000 | 0.0009 | 0.0000 |
| SULCV | 0.0660 | 0.0941 | 0.0875 | 0.0039 | 0.0000 | 0.1121 | 0.0000 | 0.0002 | 0.0000 |
| AULDI | 0.0217 | 0.0584 | 0.1829 | 0.0000 | 0.0000 | 0.0115 | 0.0000 | 0.0000 | 0.0000 |
| AULDII | 0.0269 | 0.0303 | 0.1701 | 0.0000 | 0.0000 | 0.0123 | 0.0000 | 0.0000 | 0.0000 |
| AULDIII | 0.0416 | 0.0309 | 0.1013 | 0.0002 | 0.0000 | 0.0094 | 0.0000 | 0.0001 | 0.0001 |
| AULDIV | 0.0368 | 0.0313 | 0.0919 | 0.0002 | 0.0000 | 0.0265 | 0.0000 | 0.0000 | 0.0000 |
| AULDV | 0.0402 | 0.0480 | 0.0731 | 0.0000 | 0.0000 | 0.0149 | 0.0000 | 0.0000 | 0.0000 |
| AULWI | 0.0220 | 0.0409 | 0.2369 | 0.0000 | 0.0000 | 0.0200 | 0.0000 | 0.0000 | 0.0000 |
| AULWII | 0.0541 | 0.0466 | 0.1584 | 0.0000 | 0.0000 | 0.0103 | 0.0000 | 0.0000 | 0.0000 |
| AULWIII | 0.0447 | 0.0451 | 0.1778 | 0.0000 | 0.0000 | 0.0203 | 0.0000 | 0.0000 | 0.0000 |
| AULWIV | 0.0796 | 0.0799 | 0.1677 | 0.0000 | 0.0000 | 0.0089 | 0.0000 | 0.0000 | 0.0000 |
| AULWV | 0.0615 | 0.0396 | 0.0666 | 0.0000 | 0.0000 | 0.0141 | 0.0000 | 0.0000 | 0.0000 |
| AULCI | 0.0220 | 0.0598 | 0.2019 | 0.0001 | 0.0000 | 0.0260 | 0.0000 | 0.0002 | 0.0001 |
| AULCII | 0.0529 | 0.0518 | 0.1908 | 0.0002 | 0.0000 | 0.0289 | 0.0000 | 0.0000 | 0.0001 |
| AULCIII | 0.0604 | 0.0595 | 0.1471 | 0.0014 | 0.0000 | 0.0450 | 0.0000 | 0.0000 | 0.0005 |
| AULCIV | 0.0602 | 0.0936 | 0.1300 | 0.0013 | 0.0000 | 0.0600 | 0.0000 | 0.0002 | 0.0000 |
| AULCV | 0.1260 | 0.0811 | 0.0961 | 0.0002 | 0.0000 | 0.0572 | 0.0000 | 0.0000 | 0.0000 |
| WILDI | 0.0374 | 0.1040 | 0.1110 | 0.0025 | 0.0000 | 0.0483 | 0.0000 | 0.0000 | 0.0000 |
| WILDII | 0.0393 | 0.0551 | 0.1224 | 0.0004 | 0.0000 | 0.0658 | 0.0000 | 0.0000 | 0.0001 |
| WILDIII | 0.0546 | 0.0504 | 0.0895 | 0.0002 | 0.0000 | 0.0670 | 0.0000 | 0.0000 | 0.0000 |
| WILDIV | 0.0639 | 0.0569 | 0.0691 | 0.0001 | 0.0000 | 0.0390 | 0.0000 | 0.0002 | 0.0000 |
| WILDV | 0.0814 | 0.0883 | 0.0731 | 0.0003 | 0.0000 | 0.0530 | 0.0000 | 0.0000 | 0.0000 |
| WILWI | 0.0226 | 0.0467 | 0.2035 | 0.0004 | 0.0000 | 0.0255 | 0.0000 | 0.0000 | 0.0000 |
| WILWII | 0.0178 | 0.0548 | 0.2644 | 0.0023 | 0.0000 | 0.0215 | 0.0000 | 0.0000 | 0.0000 |
| WILWIII | 0.0647 | 0.1113 | 0.1898 | 0.0003 | 0.0000 | 0.0284 | 0.0000 | 0.0000 | 0.0000 |
| WILWIV | 0.0945 | 0.1793 | 0.0847 | 0.0001 | 0.0000 | 0.0189 | 0.0000 | 0.0000 | 0.0000 |
| WILWV | 0.0875 | 0.0917 | 0.1066 | 0.0002 | 0.0000 | 0.0559 | 0.0000 | 0.0000 | 0.0000 |
| WILCI | 0.0573 | 0.1839 | 0.1413 | 0.0024 | 0.0000 | 0.0954 | 0.0000 | 0.0000 | 0.0007 |
| WILCII | 0.0794 | 0.1618 | 0.1169 | 0.0004 | 0.0000 | 0.1865 | 0.0000 | 0.0001 | 0.0003 |
| WILCIII | 0.0751 | 0.1466 | 0.1181 | 0.0003 | 0.0000 | 0.1230 | 0.0000 | 0.0001 | 0.0001 |
| WILCIV | 0.1399 | 0.1834 | 0.0864 | 0.0001 | 0.0000 | 0.0863 | 0.0000 | 0.0002 | 0.0002 |
