# Supplementary material for: Hydrological and soil physiochemical variables determine the rhizospheric microbiota in subtropical lakeshore areas
Source: PeerJ. 2020 Sep 29;8:e10078. doi: 10.7717/peerj.10078 (PMC7531358; doi:10.7717/peerj.10078)
Supplement: Table S3 [file peerj-08-10078-s003.docx]

**Table S3.** Relationships between hydrological and soil physiochemical variables and relative abundances of dominant OTUs.

| Variable (y) | OTU (x) | OTU taxa | Pearson correlation coefficient | Regression equation | a (mean ± Std. Error) | b (mean ± Std. Error) | R^2^ | t value for a | t value for b | p value for a | p value for b |
| --- | --- | --- | --- | --- | --- | --- | --- | --- | --- | --- | --- |
| TN | OTU119 | f_0319-6A21 | 0.365 | y = a × log_10_(100x+1)+b | 2.426 ± 0.782 | 0.843 ± 0.076 | 0.133 | 3.103 | 11.04 | 0.003 | 1.12 × 10^-15^ |
| TP | OTU18 | f_Rhodospirillaceae | 0.379 | y = a × x + b | 57.704 ± 18.814 | 0.383 ± 0.067 | 0.144 | 3.067 | 5.681 | 0.0003 | 4.98 × 10^-7^ |
| pH | OTU18 | f_Rhodospirillaceae | 0.529 | y = a × log_10_(100x+1)+b | -3.828 ± 0.4132 | 7.845 ± 0.170 | 0.280 | -9.265 | 46.098 | 6.76 × 10^-13^ | < 2 × 10^-16^ |
|  | OTU32 | f_Acidobacteriaceae_(Subgroup_1) | -0.618 | y = a × log_10_(100x+1)+b | -3.981 ± 0.586 | 7.586 ± 0.190 | 0.382 | -6.79 | 39.91 | 7.69 × 10^-9^ | < 2 × 10^-16^ |
|  | OTU12 | g_Candidatus_Koribacter | -0.522 | y = a × log_10_(100x+1)+b | -3.485 ± 0.641 | 7.293 ± 0.185 | 0.273 | -5.438 | 39.343 | 1.22 × 10^-6^ | < 2 × 10^-16^ |
|  | OTU19 | c_Acidobacteria(Subgroup_2) | -0.570 | y = a × log_10_(100x+1)+b | -4.628 ± 0.660 | 7.388 ± 0.166 | 0.325 | -7.011 | 44.438 | 3.32 × 10^-9^ | < 2 × 10^-16^ |
|  | OTU38 | g_Candidatus_Solibacter | -0.451 | y = a × log_10_(100x+1)+b | -4.831 ± 1.074 | 7.338 ± 0.216 | 0.203 | -4.498 | 34.053 | 3.51 × 10^-5^ | < 2 × 10^-16^ |
|  | OTU5 | f_Nitrospiraceae | -0.358 | y = a × log_10_(100x+1)+b | -4.162 ± 1.073 | 7.197 ± 0.211 | 0.128 | -3.879 | 34.068 | 0.0003 | < 2 × 10^-16^ |
|  | OTU7 | f_Acidobacteriaceae_(Subgroup_1) | -0.649 | y = a × log_10_(100x+1)+b | -6.435 ± 0.834 | 7.498 ± 0.165 | 0.422 | -7.721 | 45.520 | 2.23 × 10^-10^ | < 2 × 10^-16^ |
|  | OTU16894 | f_Acidobacteriaceae_(Subgroup_1) | -0.657 | y = a × log_10_(100x+1)+b | -6.763 ± 0.894 | 7.501 ± 0.168 | 0.431 | -7.561 | 44.787 | 4.09 × 10^-10^ | < 2 × 10^-16^ |
|  | OTU5510 | c_Acidobacteria(Subgroup_2) | -0.565 | y = a × log_10_(100x+1)+b | -5.337 ± 0.935 | 7.234 ± 0.174 | 0.320 | -5.707 | 41.685 | 4.54 × 10^-7^ | < 2 × 10^-16^ |
|  | OTU11 | f_Acidobacteriaceae_(Subgroup_1) | -0.575 | y = a × log_10_(100x+1)+b | -5.984 ± 1.012 | 7.284 ± 0.175 | 0.330 | -5.915 | 41.628 | 2.09 × 10^-7^ | < 2 × 10^-16^ |
|  | OTU8 | f_Acidobacteriaceae_(Subgroup_1) | -0.491 | y = a × log_10_(100x+1)+b | -6.520 ± 1.189 | 7.379 ± 0.195 | 0.241 | -5.485 | 37.865 | 1.03 × 10^-6^ | < 2 × 10^-16^ |
|  | OTU46 | o_Sva0485 | -0.429 | y = a × log_10_(100x+1)+b | -4.888 ± 1.118 | 7.155 ± 0.191 | 0.184 | -4.373 | 37.408 | 5.39 × 10^-5^ | < 2 × 10^-16^ |
|  | OTU29 | f_FW13 | -0.331 | y = a × log_10_(100x+1)+b | -6.901 ± 1.488 | 7.386 ± 0.219 | 0.109 | -4.639 | 33.773 | 2.15 × 10^-5^ | < 2 × 10^-16^ |
|  | OTU3053 | f_Acidobacteriaceae_(Subgroup_1) | -0.386 | y = a × log_10_(100x+1)+b | -5.395 ± 1.182 | 7.140 ± 0.185 | 0.149 | -4.563 | 38.586 | 2.8 × 10^-5^ | < 2 × 10^-16^ |
|  | OTU23 | c_Acidobacteria(Subgroup_2) | -0.396 | y = a × log_10_(100x+1)+b | -5.516 ± 1.258 | 7.125 ± 0.187 | 0.157 | -4.384 | 38.131 | 5.18 × 10^-5^ | < 2 × 10^-16^ |
|  | OTU28 | f_Nitrospiraceae | 0.469 | y = a × 10^x^+b | 90.26 ± 22.74 | -84.11 ± 22.86 | 0.220 | 3.969 | -3.679 | 0.0002 | 0.0005 |
|  | OTU152 | f_Acidobacteriaceae_(Subgroup_1) | -0.626 | y = a × log_10_(100x+1)+b | -8.875 ± 1.346 | 7.329 ± 0.168 | 0.392 | -6.593 | 43.732 | 1.62 × 10^-8^ | < 2 × 10^-16^ |
|  | OTU156 | f_Acidobacteriaceae_(Subgroup_1) | -0.452 | y = a × log_10_(100x+1)+b | -8.751 ± 1.862 | 7.332 ± 0.209 | 0.204 | -4.699 | 35.135 | 1.74 × 10^-5^ | < 2 × 10^-16^ |
| Moisture | OTU9 | o_43F-1404R | 0.402 | y = a × log_10_(100x+1)+b | 17.868 ± 4.946 | 21.187 ± 1.281 | 0.161 | 3.612 | 16.540 | 0.0007 | 2 × 10^-16^ |
|  | OTU12926 | o_Sva0485 | 0.392 | y = a × log_10_(100x+1)+b | 33.514 ± 8.728 | 21.399 ± 1.199 | 0.154 | 3.84 | 17.84 | 0.0003 | < 2 × 10^-16^ |
|  | OTU293 | p_Bathyarchaeota | 0.331 | y = a × log_10_(100x+1)+b | 35.884 ± 12.770 | 22.139 ± 1.252 | 0.110 | 2.81 | 17.69 | 0.007 | < 2 × 10^-16^ |
|  | OTU115 | g_Sphingomonas | -0.449 | y = a × log_10_(100x+1)+b | -47.111 ± 11.326 | 27.176 ± 1.108 | 0.201 | -4.159 | 24.536 | 0.0001 | < 2 × 10^-16^ |
|  | OTU295 | g_RB41 | -0.281 | y = a × log_10_(100x+1)+b | -33.55 ± 13.24 | 25.49 ± 1.05 | 0.079 | -2.533 | 24.269 | 0.014 | < 2 × 10^-16^ |
|  | OTU1143 | g_Geobacter | 0.523 | y = a × log_10_(100x+1)+b | 75.270 ± 15.500 | 21.856 ± 1.003 | 0.274 | 4.856 | 21.784 | 1 × 10^-5^ | < 2 × 10^-16^ |
| FRWL | OTU3 | f_Acidobacteriaceae_(Subgroup_1) | 0.809 | y = a × log_10_(100x+1)+b | 302.24 ± 67.55 | 83.38 ± 27.82 | 0.253 | 4.475 | 2.997 | 3.8 × 10^-5^ | 0.004 |
|  | OTU32 | f_Acidobacteriaceae_(Subgroup_1) | 0.763 | y = a × log_10_(100x+1)+b | 290.17 ± 86.45 | 109.58 ± 28.03 | 0.189 | 3.357 | 3.910 | 0.001 | 0.0003 |
|  | OTU12 | g_Candidatus_Koribacter | 0.796 | y = a × log_10_(100x+1)+b | 360.00 ± 81.69 | 111.16 ± 23.63 | 0.232 | 4.407 | 4.704 | 4.79 × 10^-5^ | 1.71 × 10^-5^ |
|  | OTU19 | c_Acidobacteria(Subgroup_2) | 0.816 | y = a × log_10_(100x+1)+b | 496.67 ± 85.54 | 98.28 ± 21.54 | 0.272 | 5.806 | 4.562 | 3.13 × 10^-7^ | 2.81 × 10^-5^ |
|  | OTU5510 | c_Acidobacteria(Subgroup_2) | 0.823 | y = a × log_10_(100x+1)+b | 597.18 ± 115.94 | 112.17 ± 21.52 | 0.282 | 5.151 | 5.213 | 3.48 × 10^-6^ | 2.77 × 10^-6^ |
|  | OTU11 | f_Acidobacteriaceae_(Subgroup_1) | 0.794 | y = a × log_10_(100x+1)+b | 595.04 ± 132.29 | 114.58 ± 22.88 | 0.250 | 4.498 | 5.008 | 3.50 × 10^-5^ | 5.81 × 10^-6^ |
|  | OTU152 | f_Acidobacteriaceae_(Subgroup_1) | 0.797 | y = a × log_10_(100x+1)+b | 771.29 ± 188.35 | 118.72 ± 23.45 | 0.231 | 4.095 | 5.063 | 0.0001 | 4.78 × 10^-6^ |
|  | OTU13 | c_SAGMCG-1 | 0.647 | y = a × log_10_(100x+1)+b | 468.09 ± 142.73 | 153.81 ± 20.59 | 0.115 | 3.279 | 7.469 | 0.002 | 5.82 × 10^-10^ |
|  | OTU864 | f_Acidobacteriaceae_(Subgroup_1) | 0.452 | y = a × log_10_(100x+1)+b | 683.10 ± 189.03 | 137.65 ± 21.97 | 0.147 | 3.614 | 6.265 | 0.0006 | 5.62 × 10^-8^ |
|  | OTU5738 | f_Acidobacteriaceae_(Subgroup_1) | 0.796 | y = a × log_10_(100x+1)+b | 846.27 ± 183.72 | 127.49 ± 20.98 | 0.238 | 4.606 | 6.077 | 2.41 × 10^-5^ | 1.14 × 10^-7^ |
|  | OTU45 | g_Candidatus_Solibacter | 0.824 | y = a × log_10_(100x+1)+b | 883.91 ± 206.33 | 122.65 ± 22.35 | 0.239 | 4.284 | 5.487 | 7.28 × 10^-5^ | 1.02 × 10^-6^ |
|  | OTU293 | p_Bathyarchaeota | -0.873 | y = a × log_10_(100x+1)+b | -816.21 ± 258.99 | 230.41 ± 25.39 | 0.140 | -3.152 | 9.075 | 0.003 | 1.36 × 10^-12^ |
|  | OTU50 | g_Candidatus_Methanoperedens | -0.829 | y = a × log_10_(100x+1)+b | -404.47 ± 169.87 | 196.74 ± 21.39 | 0.088 | -2.381 | 9.200 | 0.021 | 8.6 × 10^-13^ |
