# Supplementary figures and images for: Hydrological and soil physiochemical variables determine the rhizospheric microbiota in subtropical lakeshore areas
Source: PeerJ. 2020 Sep 29;8:e10078. doi: 10.7717/peerj.10078 (PMC7531358; doi:10.7717/peerj.10078)

(A)

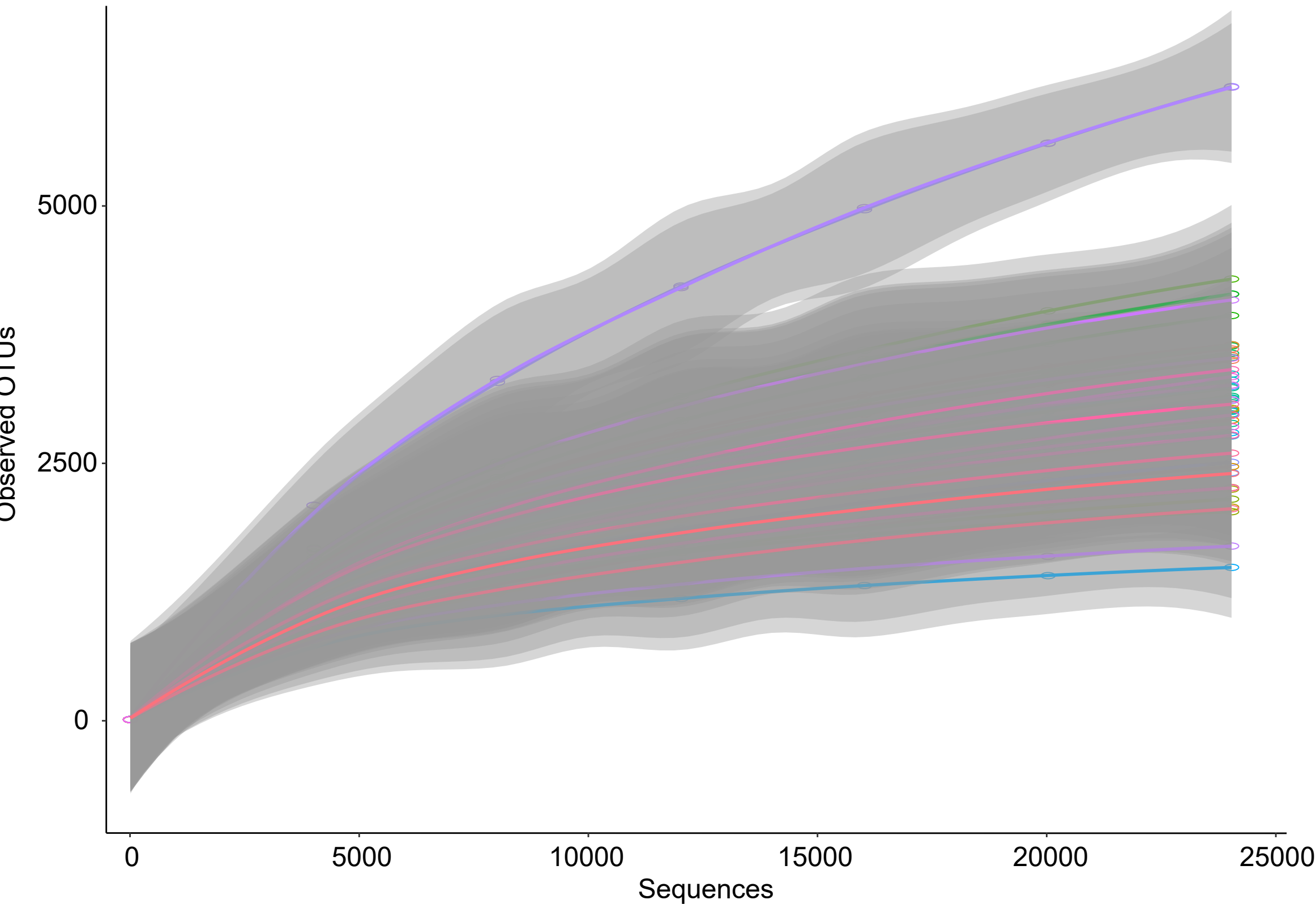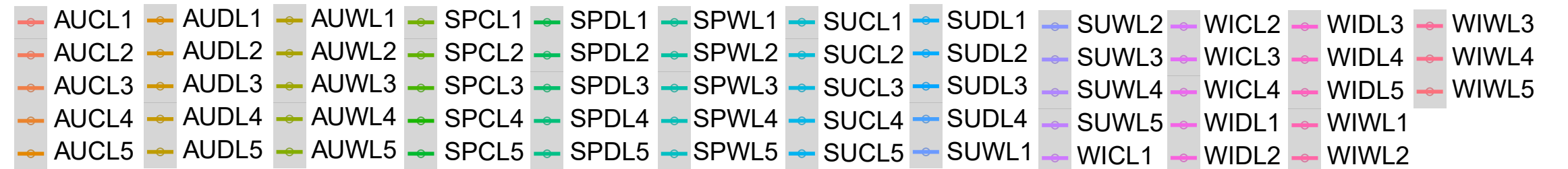

(B)

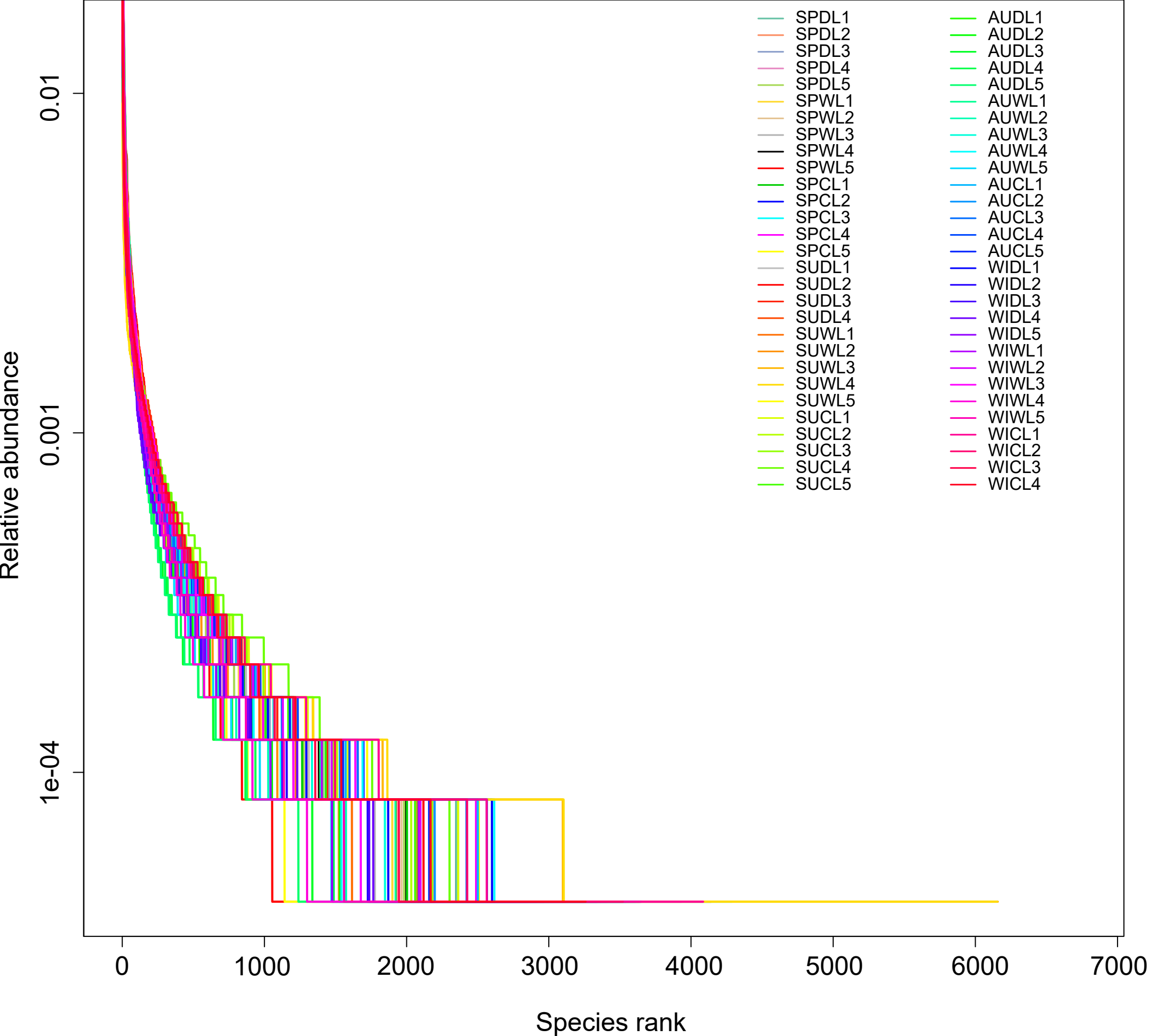

Supplement: Figure S1 — Along each transect, five sites numbered I, II, III, IV, and V were sequentially set perpendicular to the lakeshore from the mean annual lowest water level to its highest water level. The elevation differences were equal between any two adjacent sites in each transect. The sample group names were formed by combining sampling season, lake, and sampling site. AU, autumn; LD, Lake Dahuchi; hence AULDIII indicated the sample taken from the III site of Lake Dahuchi in autumn, 2016. [file peerj-08-10078-s004.pdf]

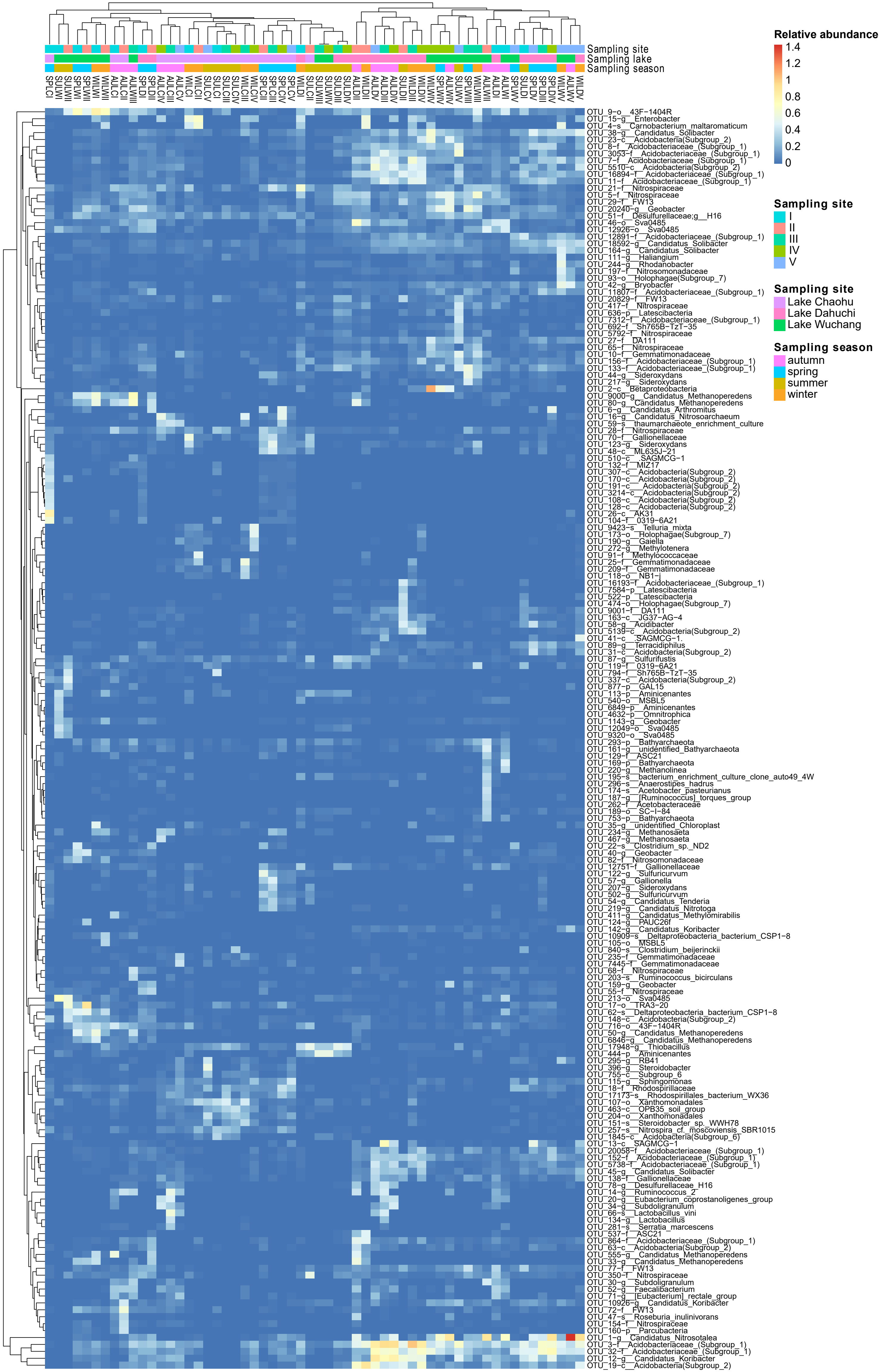

Supplement: Figure S2 — SP, spring; SU, summer; AU, autumn; WI, winter; LC, Lake Chaohu; LW, Lake Wuchang; LD, Lake Dahuchi. Therefore, for example, SPLWIII indicated the sample taken from the III site of Lake Wuchang in spring, 2017. [file peerj-08-10078-s005.pdf]

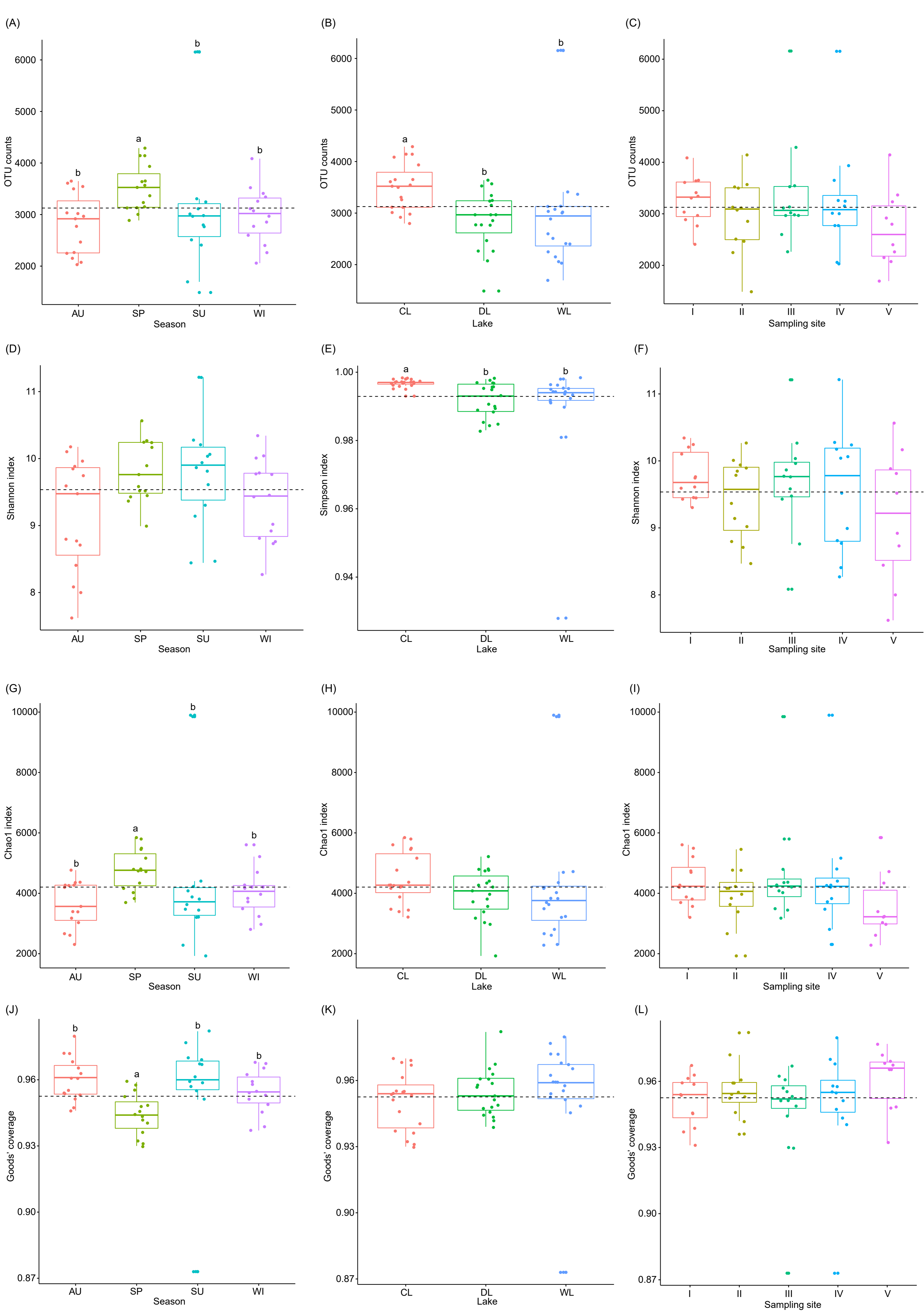

Supplement: Figure S3 [file peerj-08-10078-s006.pdf]

(A)

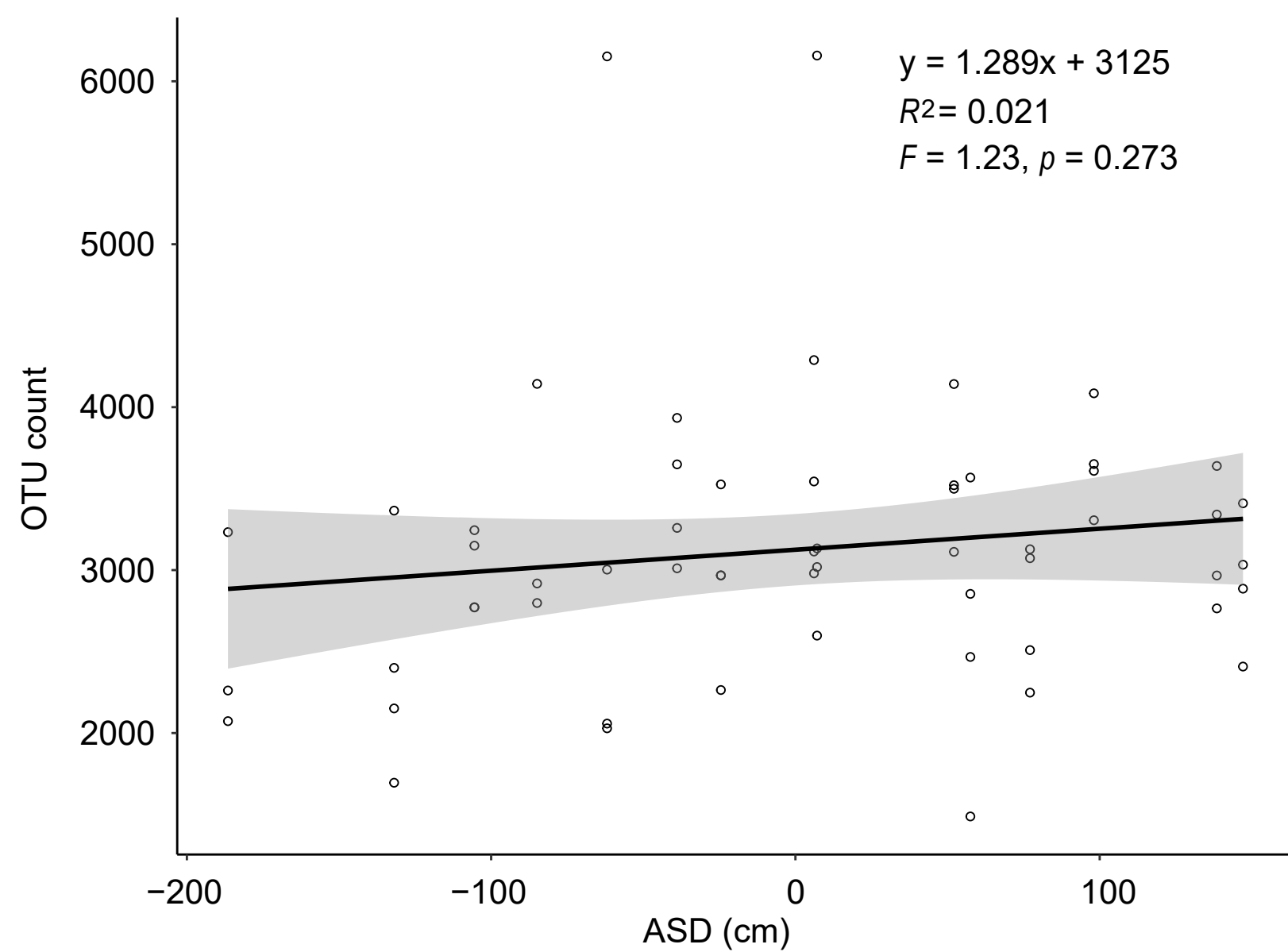

(B)

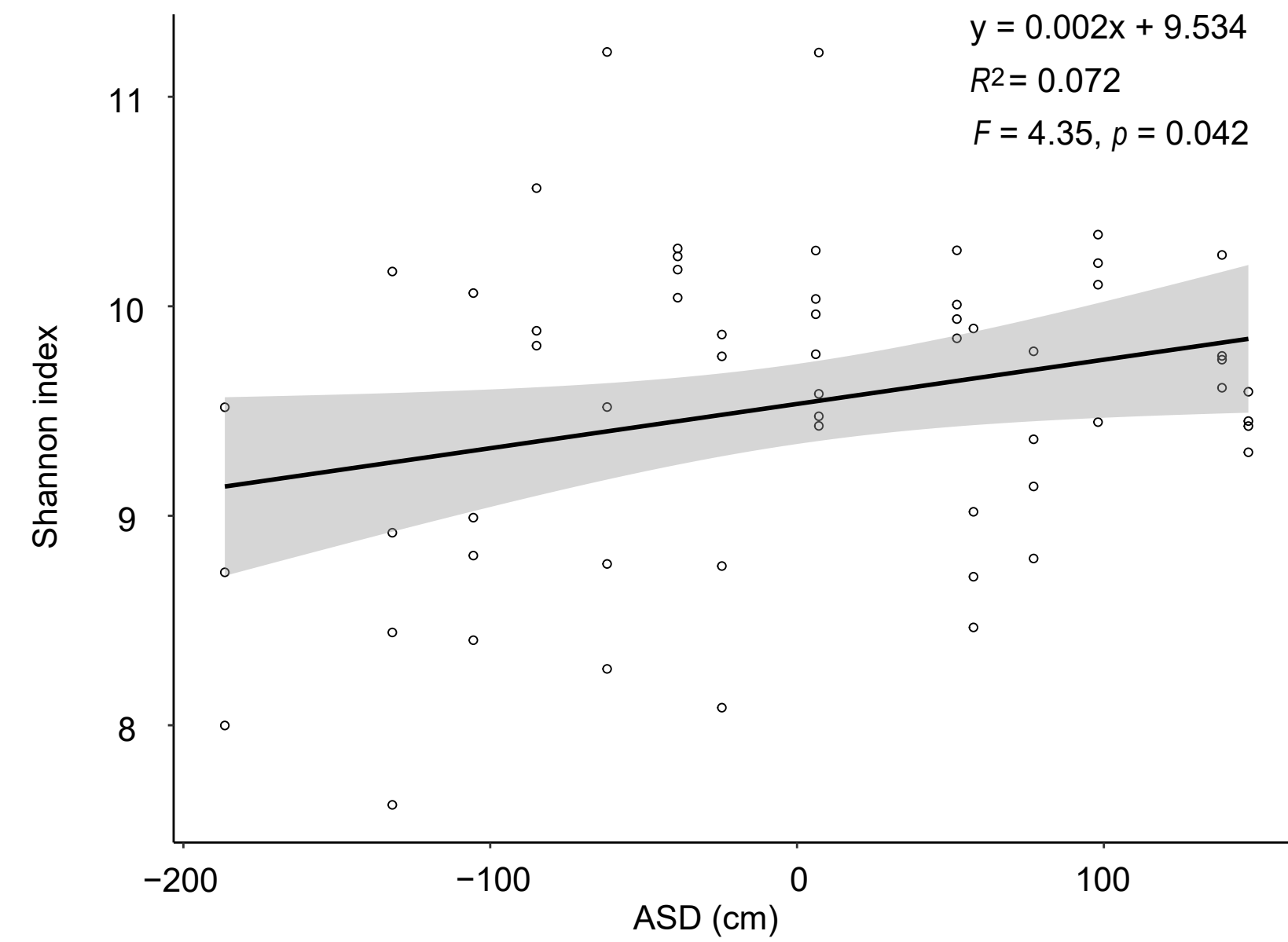

(C)

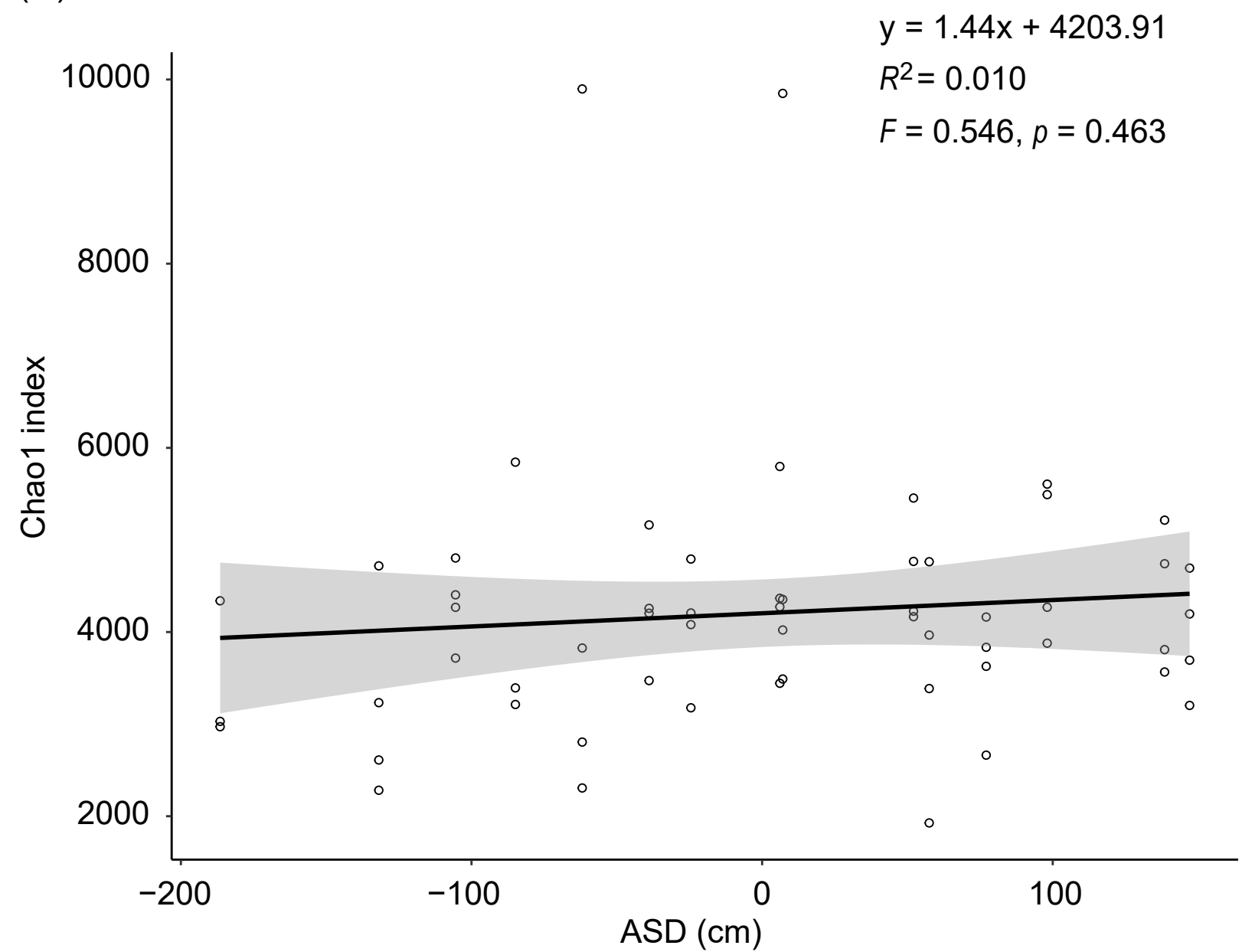

(D)

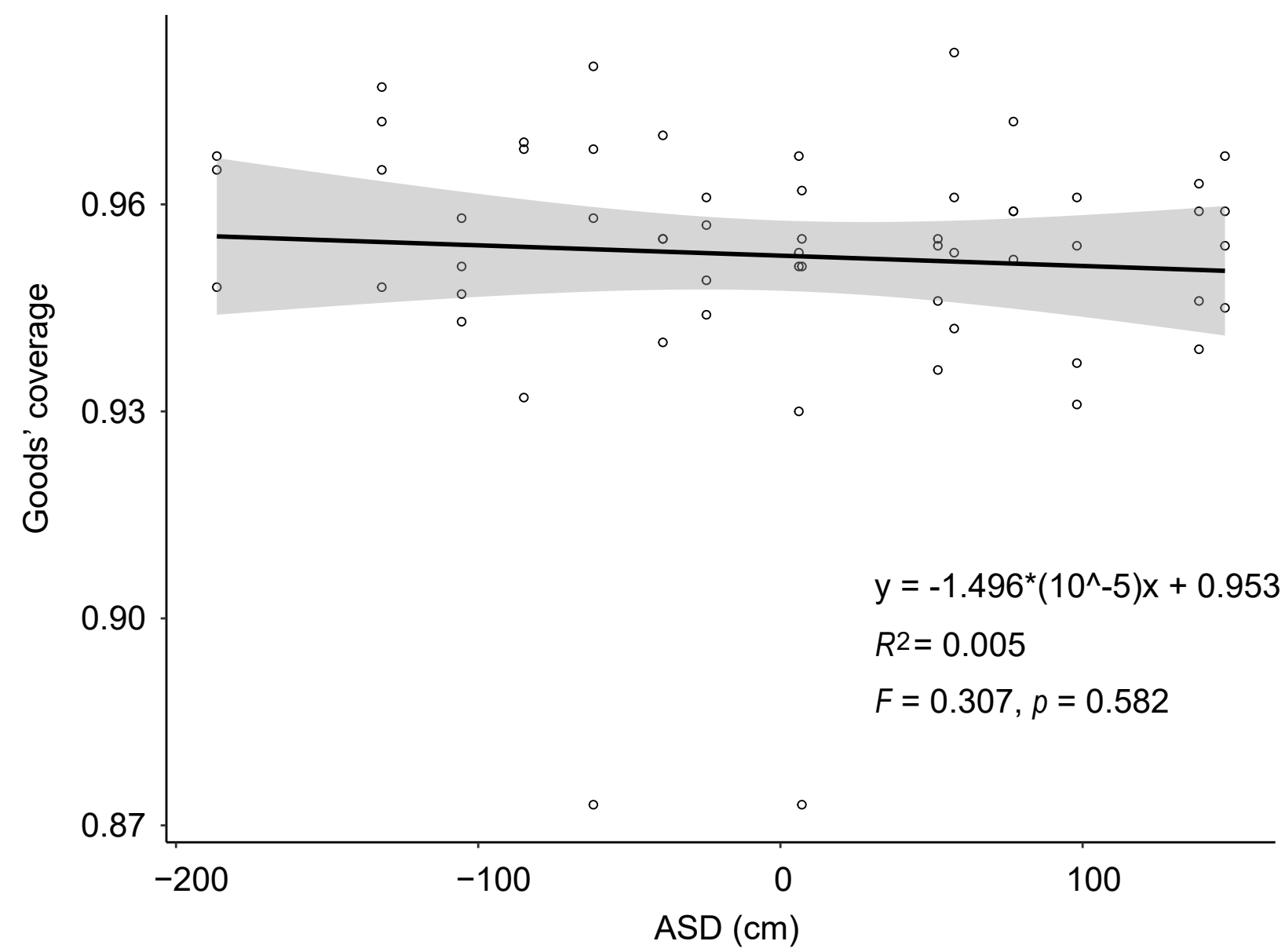

Supplement: Figure S4 [file peerj-08-10078-s007.pdf]

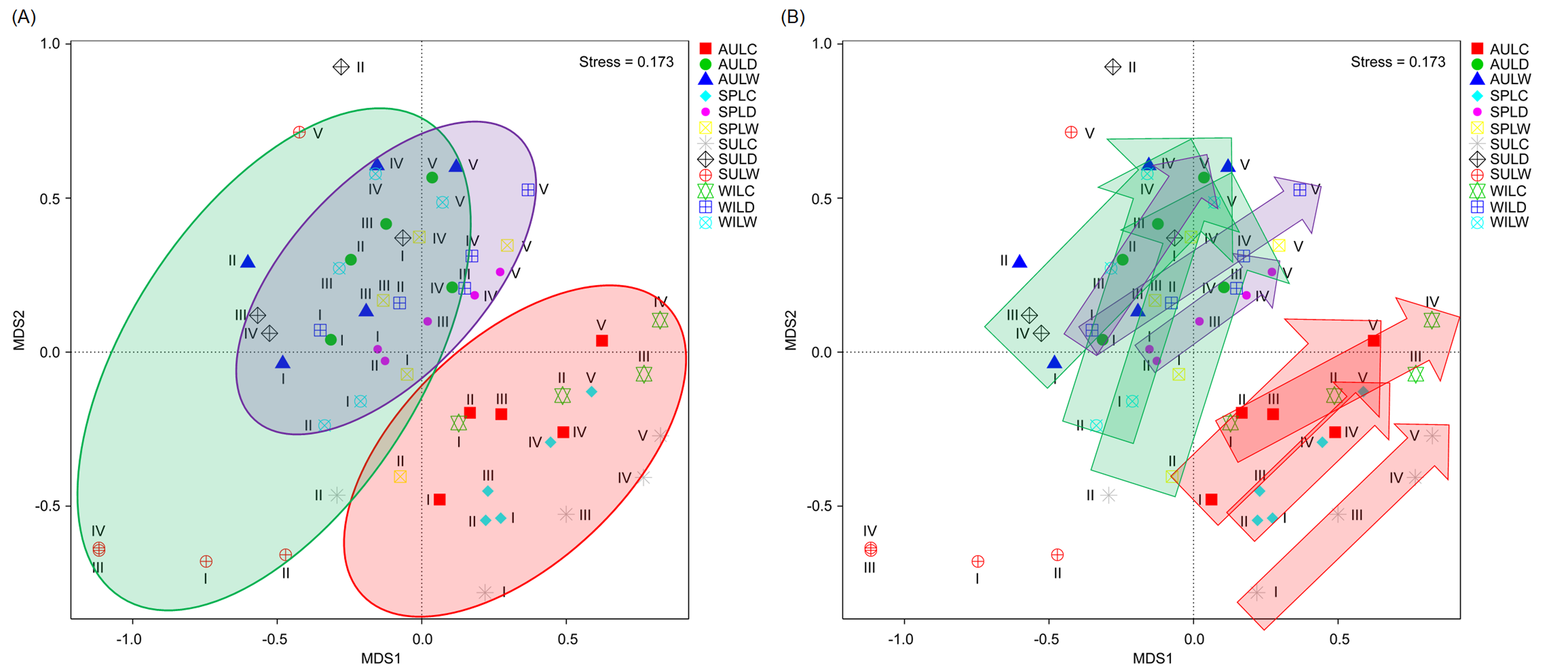

Supplement: Figure S5 — The arrows indicate the trend from sampling site I to sampling site V. The sample group names were formed by combining sampling season, lake, and sampling site. AU, autumn; LD, Lake Dahuchi; hence AULDIII indicated the sample taken from the III site of Lake Dahuchi in autumn, 2016. [file peerj-08-10078-s008.png]

Pearson correlation matrix

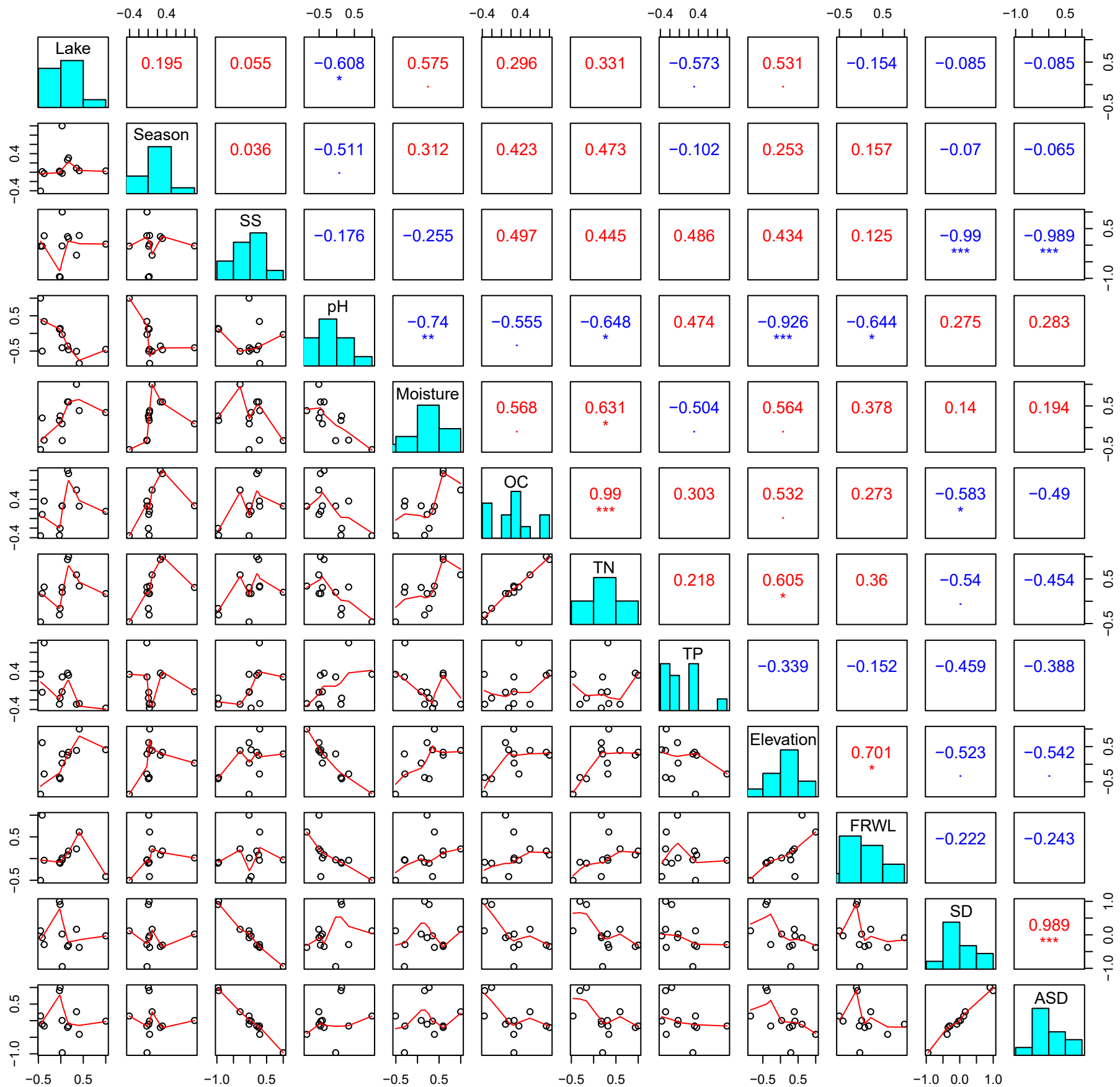

Supplement: Figure S6 — The values in the upper right of the picture were the Pearson correlation coefficients between the environmental factors corresponding to the row and column of the values. Red and blue values indicate positive and negative correlations, respectively. The scatter plots and line chart in each panel in the lower left of the picture showed the points on the coordinates formed by the values of the environmental factors corresponding to the row and column of the panel at each sampling point, and the correlation curve of the two environmental factors fitted according to these points. “Lake” referred the sampling lakes, i.e. Lake Chaohu, Lake Wuchang, and Lake Dahuchi; “Season” referred the sampling seasons, i.e. summer, autumn, winter, and spring. TN, total nitrogen; TP, total phosphorus; SD, submerged duration; FRWL, fluctuation range of water level; ASD, average submerged depth; OC, organic content; SS, sampling site. *, p < 0.05; **, p < 0.01; ***, p < 0.001. [file peerj-08-10078-s009.pdf]
